# Comparison of chromatin accessibility landscapes during early development of prefrontal cortex between rhesus macaque and human

**DOI:** 10.1038/s41467-022-31403-3

**Published:** 2022-07-06

**Authors:** Xuelong Yao, Zongyang Lu, Zhanying Feng, Lei Gao, Xin Zhou, Min Li, Suijuan Zhong, Qian Wu, Zhenbo Liu, Haofeng Zhang, Zeyuan Liu, Lizhi Yi, Tao Zhou, Xudong Zhao, Jun Zhang, Yong Wang, Xingxu Huang, Xiaoqun Wang, Jiang Liu

**Affiliations:** 1 CAS Key Laboratory of Genome Sciences and Information, Collaborative Innovation Center of Genetics and Development, Beijing Institute of Genomics, Chinese Academy of Sciences and China National Center for Bioinformation, 100101 Beijing, China; 2 University of Chinese Academy of Sciences, 100049 Beijing, China; 3 Guangzhou Nvwa Life Technology Co., Ltd, Guangzhou, 510535 China; 4 School of Life Science and Technology, Shanghai Tech University, 100 Haike Rd., Pudong New Area, Shanghai, 201210 China; 5 Shanghai Institute of Biochemistry and Cell Biology, Chinese Academy of Sciences, 320 Yueyang Road, Shanghai, 200031 China; 6 CEMS, NCMIS, MDIS, Academy of Mathematics and Systems Science, Chinese Academy of Sciences, 100190 Beijing, China; 7 State Key Laboratory of Brain and Cognitive Science, CAS Center for Excellence in Brain Science and Intelligence Technology, Institute of Brain-Intelligence Technology (Shanghai), Institute of Biophysics, Chinese Academy of Sciences, 100101 Beijing, China; 8 State Key Laboratory of Cognitive Neuroscience and Learning, Beijing Normal University, 100875 Beijing, China; 9 IDG/McGovern Institute for Brain Research, Beijing Normal University, 100875 Beijing, China; 10 Obstetrics and Gynecology Medical Center of Severe Cardiovascular of Beijing Anzhen Hospital, Capital Medical University, 100029 Beijing, China; 11 Shenzhen Key Lab of Drug Addiction; The Brain Cognition and Brain Disease Institute (BCBDI), Shenzhen Institutes of Advanced Technology, Chinese Academy of Sciences; Shenzhen-Hong Kong Institute of Brain Science-Shenzhen Fundamental Research Institutions, Shenzhen, 518055 China; 12 Kunming Primate Research Center, Key Laboratory of Animal Models and Human Disease Mechanisms of Chinese Academy of Sciences, Kunming Institute of Zoology, Chinese Academy of Sciences, 650223 Kunming, China; 13 Center for Excellence in Animal Evolution and Genetics, Chinese Academy of Sciences, Kunming, 650223 China; 14 Zhejiang Provincial Key Laboratory of Pancreatic Disease, The First Affiliated Hospital, and Institute of Translational Medicine, Zhejiang University School of Medicine, Hangzhou, Zhejiang 310058 China; 15 Institute for Stem Cell and Regeneration, Chinese Academy of Sciences, 100101 Beijing, China; 16 Beijing Institute for Brain Disorders, 100069 Beijing, China

**Keywords:** Neuronal development, Epigenetics and plasticity

## Abstract

Epigenetic information regulates gene expression and development. However, our understanding of the evolution of epigenetic regulation on brain development in primates is limited. Here, we compared chromatin accessibility landscapes and transcriptomes during fetal prefrontal cortex (PFC) development between rhesus macaques and humans. A total of 304,761 divergent DNase I-hypersensitive sites (DHSs) are identified between rhesus macaques and humans, although many of these sites share conserved DNA sequences. Interestingly, most of the *cis*-elements linked to orthologous genes with dynamic expression are divergent DHSs. Orthologous genes expressed at earlier stages tend to have conserved *cis*-elements, whereas orthologous genes specifically expressed at later stages seldom have conserved *cis*-elements. These genes are enriched in synapse organization, learning and memory. Notably, DHSs in the PFC at early stages are linked to human educational attainment and cognitive performance. Collectively, the comparison of the chromatin epigenetic landscape between rhesus macaques and humans suggests a potential role for regulatory elements in the evolution of differences in cognitive ability between non-human primates and humans.

## Abstract (web summary)

The evolution of epigenetic regulation of brain development in primates is not well understood. Here, the authors perform a comparative study of epigenetic dynamics of early prefrontal cortex development between human and rhesus macaque, finding divergent regulatory elements that may be related to cognitive capacity.

## Introduction

It is known that the primate brain development begins a few weeks after conception. The cellular developmental processes of the brain are largely conserved across primates, including neuron specification, migration and the formation of functional neuronal circuits^1–6^. Consequently, the anatomical structures and developmental procedures for primate brains are well matched^7–9^. In addition, transcriptional maps of primate brain development have been widely studied recently^9–11^. Human and non-human primates (NHPs) share similar patterns of gene expression, revealing that the underlying gene networks regulating primate brain development are also conserved. However, during the primate evolution, the cognitive capacity of the brain is dramatically different between humans and NHPs^12,13^. In particular, human beings have acquired a series of ‘higher’ cognitive functions, such as language, social interaction and problem solving. Unfortunately, our knowledge of what contributes to the differences in cognitive capacity between humans and NHPs is limited.

Previous studies have proven that the prefrontal cortex (PFC) plays important roles in cognition and behavior, which is responsible for the ‘higher’ cognitive functions in humans^14,15^. Dysfunction of the PFC leads to cognitive abnormalities and nervous system diseases^16,17^. Thus, the investigation of the molecular mechanisms involved in the functions of the PFC can provide us insight into cognitive functions. Many lines of evidence support that the genes and regulatory mechanisms participating in brain development are critical for the normal functions of the brain. Many neuropsychiatric diseases associated with genomic variants are located in the brain development-related genes or regulatory elements^18,19^. Thus, a comparative study of the underlying mechanisms regulating PFC development in primates would explain the cognitive diversity between humans and NHPs.

Epigenetic information can regulate gene expression by influencing the chromatin state in the *cis*-elements^20,21^. Activation of *cis*-elements such as enhancers and promoters can drive the expression of genes that determine cell fate^22^. These active *cis*-elements are usually located at open chromatin regions that are preferentially occupied by transcription factors^23^. Mutations in *cis*-elements can affect the epigenetic states and the expression levels of associated genes^24,25^. Genomic sequencing can be utilized to compare the gene expression patterns and chromatin states in the PFC across species. Recently, significant advances in understanding the evolution of the PFC have been achieved through the comparison of gene expression across different species^26^. However, the impact of the chromatin states of *cis*–elements on PFC development during primate evolution is very limited known. The DNase I-hypersensitive site sequencing (DNase-seq) method to map chromatin accessibility is usually applied to identify regulatory elements in the genome^27,28^. The rhesus monkey is a widely used NHP model to study the nerve system development and diseases, and is also a good model in understanding the conservation and divergence of the chromatin states of *cis*-elements in comparison with humans.

In this work, we compared the chromatin accessibility landscape between rhesus and human PFC development. Overall chromatin accessibility and gene expression are conserved between rhesus monkeys and humans, which is consistent with PFC development. Many *cis*-elements with conserved sequences show divergent chromatin accessibility states between rhesus monkeys and humans. Orthologous genes with conserved DHSs tend to be expressed in the PFC at earlier stages, while orthologous genes specifically expressed at later stages mainly harbor divergent DHSs. DHSs in the PFC at earlier stages are linked to human intelligence associated activities. Our evolutionary comparison advances our knowledge of the differences in cognitive capacity between humans and rhesus monkeys.

## Results

### The conservation of the regulatory landscape between rhesus monkeys and humans during PFC development

**Fig. 1:**
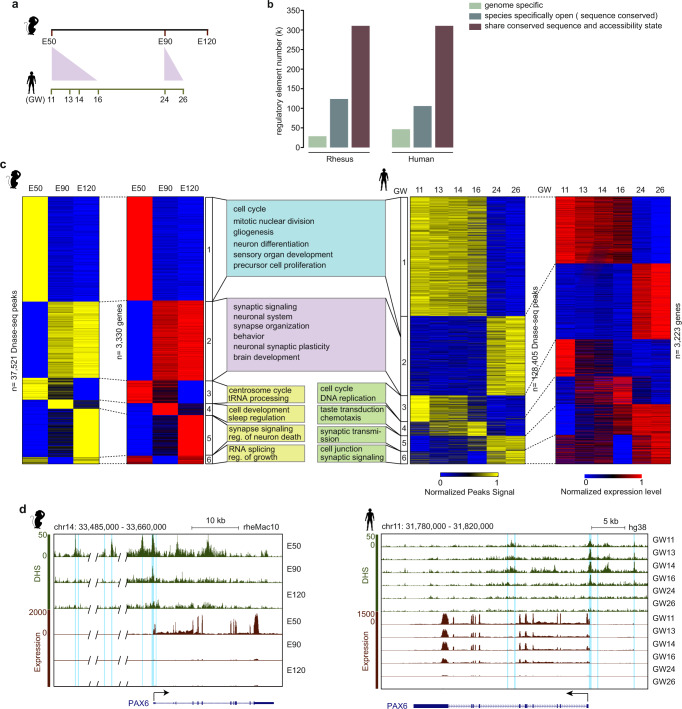
Dynamics of the chromatin accessibility landscape during human and rhesus PFC development. **a** Schematic of developmental stages examined in rhesus monkeys and humans. **b** The number of conserved and divergent regulatory elements between rhesus and human PFCs. **c** The dynamics of gene expression and the DHS signal of paired regulatory elements in rhesus (left) and human (right) PFC. The potential regulatory element-gene pairs are classified into 6 clusters by the K-means method. The middle panel shows the significantly enriched GO terms. Reg. represents regulation. In cluster 6, all the DHSs assigned to the orthologous genes, in addition to the DHSs detected separating the gene activation at different stages, are shown. **d** Genome browser view of the expression of *PAX6* and the DHS signal of the paired regulatory elements during PFC development in rhesus monkeys (left) and humans (right). The light blue shadows mark the paired regulatory elements.

To compare the epigenetic regulation of PFC development between rhesus monkeys and humans, we mapped chromatin accessibility landscapes at different embryonic stages for both rhesus monkeys and humans by using the DNase-seq method (Supplementary Fig. 1a, b). We collected rhesus PFC at embryonic day 50 (E50), E90, E120 and human PFC at the gestational week (GW) 11, GW13, GW14, GW16, GW24, and GW26 stages (Fig. 1a). For the developmental stages of PFCs, rhesus E50 is equivalent to human GW11-GW16, while rhesus E90 is equivalent to human GW24-GW26. For each embryonic stage, two samples were sequenced. The replicates for each stage were highly reproducible (Supplementary Fig. 1c, d). Consistent with previous reports^29,30^, DHSs were highly enriched in promoters across all stages in both rhesus monkeys and humans (Supplementary Fig. 2a, b). A previous study identified active enhancers and promoters during human and rhesus corticogenesis according to histone modifications^31^. Approximately 50% of the enhancers and promoters identified in that study overlapped with the DHSs identified in our data (Supplementary Fig. 2c, d). Collectively, our DNase-seq data are robust and reliable. By comparing the DNA sequence and chromatin state of *cis*-elements between rhesus monkeys and humans, we identified 310,445 pairs of *cis*-elements with conserved sequences that are both open in humans and rhesus monkeys, while there are 304,761 divergent DHSs between rhesus monkeys and humans. Moreover, among these divergent *cis*-elements, 105,785 *cis*-elements with conserved DNA sequences are only open in humans but not in rhesus monkeys; 123,664 *cis*-elements with conserved sequences are only open in rhesus monkeys but not in humans; 46,603 *cis*-elements with open chromatin states in humans do not have conserved sequences in rhesus monkeys; and 28,709 sequences with open chromatin states in rhesus monkeys do not have conserved sequences in humans (see method; Fig. 1b)

To explore the regulatory functions of *cis*-elements during human and rhesus PFC development, we performed RNA-seq in PFC samples (Supplementary Fig. 3a, b). Consistent with previous reports, genes with promoter DHSs show significantly higher expression levels than genes without promoter DHSs (Supplementary Fig. 3c, d). During PFC development, a large number of genes show dynamic expression. Next, we identified the potential regulatory elements for these genes based on the correlation between gene expression and DHS signals (within 1 Mb around the transcription start site) at different stages^32^. The DHS, whose signal is highly correlated with the expression of a gene (Pearson’s coefficient > 0.8, *P* value < 0.05), is referred to as the paired regulatory element for the gene (Supplementary Fig. 4a). In total, we obtained the paired regulatory elements for 3330 dynamic genes and 3223 dynamic genes in rhesus monkeys and humans, respectively (Fig. 1c, Supplementary Table 1 and Supplementary Table 2). More paired regulatory elements were identified in humans than rhesus monkeys, which may be mainly attributed to the larger sample size investigated in humans than rhesus monkeys. We further performed K-means clustering and gene ontology (GO) analyses on the regulatory element-gene pairs (Fig. 1c, Supplementary Data 1). Our data show that genes with paired regulatory elements, which are mainly expressed at the rhesus E50 stage, are enriched in the cell cycle, neuron differentiation and brain development (Fig. 1c cluster 1, Supplementary Data 1). Similar enriched categories can also be observed in the genes that are mainly expressed at human early stages (GW11, 13, 14, 16) (Fig. 1c cluster 1 in the right panel). For example, the dynamics of the chromatin accessibility states of several *cis*-elements are consistent with the expression dynamics of *PAX6* (Fig. 1d). Moreover, the genes in cluster 1 are also enriched in the immune-related pathways (such as RHO GTPase effectors and signaling by Rho GTPases) and human cytomegalovirus (HCMV)-associated biological processes (such as early HCMV events, late HCMV events and HCMV infection) (Supplementary Data 1). Consistently, previous studies have proven that Rho GTPases play essential roles in immunoreaction and neurological disorders such as autism^33–35^, and HCMV affects the neuronal progenitor cell proliferation and differentiation^36,37^. Similar GO enrichment can also be found between the genes that are mainly expressed at the rhesus E90 and E120 stages and the genes that are expressed at the human GW24 and 26 stages (Fig. 1c). Genes in rhesus cluster 2 and genes in human cluster 2 are both enriched in synapse organization, regulation of neuronal synaptic plasticity and neuron transmitter transport, consistent with that synaptic development takes place at the corresponding stages in rhesus and human^38^. For example, the synaptic-related gene *SYNGR3* is specifically expressed at the rhesus E90 and E120 stages, and a paired regulatory element of this gene is specifically open at E90 and E120 (Supplementary Fig. 4b). The GO enrichments for the genes in cluster 3 to cluster 6 are not well matched between rhesus monkeys and humans (Fig. 1c). This may be caused by the fact that there is no equivalent stage to rhesus E120 in human PFC data. Taken together, the overall dynamic patterns of chromatin accessibility during PFC development are conserved between rhesus monkeys and humans, which can fit the requirements of neuronal development.

Additionally, we noticed that many genes showed low expression levels at the middle stages and high expression levels at the earlier and later stages, although the stages showing low expression levels were not equivalent between rhesus monkeys and humans (Fig.1c cluster 6). Notably, among those genes, the paired regulatory elements for some genes, including 102 rhesus genes and 350 human genes, were different between the early and late stages (Supplementary Fig. 5a). For example, in rhesus monkeys, *SLIT3* shows high expression at the E50 and E120 stages, but low expression at the E90 stage. It is potentially regulated by two different regulatory elements at the E50 and E120 stages (Supplementary Fig. 5b). The data suggest that stage-specific expression may be regulated by stage-specific *cis*-regulatory elements.

### The conservation and divergence of regulating models for orthologous genes

To investigate the impact of the epigenetic state of regulatory elements during primate PFC evolution, we focused on the comparison of orthologous genes between humans and rhesus monkeys. Our result shows that 10,902 and 10,528 orthologous genes are expressed in the PFCs of rhesus monkeys and humans, respectively. Then, we identified the potential *cis*-elements for these orthologous genes by using the correlation approach in humans and rhesus monkeys, respectively. To validate the interaction between regulatory elements and genes identified by our method, we integrated Hi-C data of the human cerebral cortex at the midgestation stages (GW17-GW18 stages) for analysis^18^. In total, 7.9% of the identified regulatory element-gene pairs are supported by the Hi-C data. In particular, there are 3223 genes with dynamic expression in human PFC, among which 1314 genes (41% of 3223 genes) have regulatory element-gene pairs that are supported by the Hi-C data (Supplementary Fig. 6). Many regulatory element-gene pairs are not detected in the Hi-C data, because the developmental stages and tissue types of the samples analyzed in our study are different from those in the Hi-C data. Furthermore, for those Hi-C validated regulatory element-gene pairs, the overall dynamic patterns of chromatin accessibility during PFC development can also fit the requirement of neuronal development, which is consistent with our results (Fig. 1c and Supplementary Fig. 6).

**Fig. 2:**
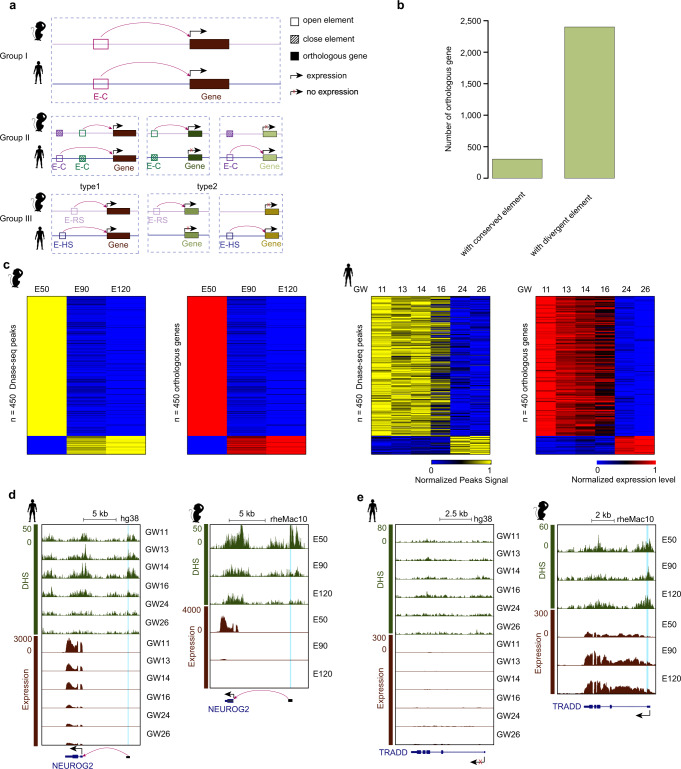
The conservation and divergence of regulating models for orthologous genes. **a** Schematics showing three groups of regulatory element-gene pairs between humans and rhesus monkeys. E–C: Element sharing a conserved sequence between human and rhesus, E-HS: element with a human-specific sequence, E-RS: element with a rhesus-specific sequence. **b** Bar plots show the number of orthologous genes with conserved or divergent elements. **c** Heatmaps show the orthologous genes with conserved regulatory elements between rhesus monkeys and humans. Each row represents a conserved regulatory element-gene pair. **d** Genome browser view of expression of the *NEUROG2* and the DHS signal of paired regulatory elements in rhesus and human PFC. The light blue shadows mark the paired regulatory elements. The paired regulatory elements of *NEUROG2* gene have conserved sequences and chromatin states between humans and rhesus monkeys. The pink arrow indicates the potential regulatory effect of the regulatory element on *NEUROG2*. **e** Genome browser track shows that *TRADD* is not expressed in the human PFC (left) but is species-specific expressed in the rhesus PFC (right). The light blue shadow marks the position of potential regulatory element of this gene in rhesus monkeys. The sequence of this element is rhesus-specific and cannot be found in humans.

Next, we investigated the conservation and divergence of the potential regulatory elements of the orthologous genes. If the paired regulatory element of a human orthologous gene shares a conserved DNA sequence and chromatin accessibility state with that of rhesus, we call this orthologous gene sharing a conserved regulatory element (see method; Fig. 2a Group I). In total, we identified 2623 conserved regulatory element-gene pairs for 2044 orthologous genes between humans and rhesus monkeys. Further transcription factor binding motif analyses show that these shared regulatory elements are enriched for binding sites of transcription factors such as NEUROG2, NEUROD1, CTCF and the homeobox transcription factor gene family (DLX1, DLX3, DLX5, LHX1, LHX2, LHX3, LHX9) (Supplementary Fig. 7a).

Then, we focused on the dynamically expressed orthologous genes. Our data show that the GO enrichments of rhesus cluster 1 and human cluster 1 are similar, and the enrichment of rhesus cluster 2 and human cluster 2 are also similar (Fig. 1c, Supplementary Data 1). These data suggest that the developmental process in rhesus monkeys from the E50 transition to E90 and E120 is consistent with the process in humans from the GW11, 13, 14, 16 transition to GW24 and 26. Therefore, we compared the orthologous genes between rhesus cluster 1 and human cluster 1, showing that 482 orthologous genes are shared in rhesus monkeys and humans, while 646 and 212 orthologous genes are specifically expressed in rhesus monkeys and humans, respectively (Supplementary Fig. 8a, Supplementary Data 2). Similarly, 391 orthologous genes are shared between rhesus cluster 2 and human cluster 2, while 533 and 440 orthologous genes are specifically expressed in rhesus monkeys and humans, respectively (Supplementary Fig. 8b, Supplementary Data 3). In contrast, GO enrichments in the other clusters are not similar between rhesus monkeys and humans, and we did not compare the orthologous genes in these clusters between rhesus monkeys and humans (Fig. 1c).

Among the dynamically expressed orthologous genes, only 304 orthologous genes have conserved paired regulatory elements (Fig. 2b). Interestingly, over 80% of these genes are mainly expressed before the GW16 stage in humans and before the E50 stage in rhesus monkeys (Fig. 2c). GO analyses show that these genes are significantly enriched in the cell cycle, forebrain development and regulation of neural precursor cell proliferation (Supplementary Fig. 8c). For example, *NEUROG2*, a neural precursor cell marker gene, is expressed before the human GW16 stage and at the rhesus E50 stage. Our data show that the paired regulatory elements of *NEUROG2* in rhesus monkeys and humans share conserved sequences and chromatin accessibility states (Fig. 2d). This is consistent with the previous work showing that the regulation of neural precursor cell proliferation among primates is conserved^5^.

However, more dynamically expressed orthologous genes have divergent paired regulatory elements between humans and rhesus monkeys (Fig. 2b). Our data reveal that approximately 45% of genes (*n* = 1083 orthologous genes) with the divergent paired regulatory elements are mainly expressed at the early PFC development stage (before human GW16 or at the rhesus E50 stage). GO analyses show that these genes are significantly enriched in cell cycle phase transition and Wnt signaling pathway (Supplementary Fig. 9a). The remaining genes (*n* = 1317 orthologous genes) are mainly expressed at the late PFC development stages (human GW24 and GW26 stages or rhesus E90 and E120 stages). GO analyses show that these genes are significantly enriched in the categories of synaptic plasticity, behavior, cognition, learning and memory (Supplementary Fig. 9b). Previous work has proven that synaptic plasticity and organization play key roles in cognition^39^. These results could provide clues to investigate the distinct capability in cognition between rhesus monkeys and humans.

Among the divergent regulatory element-gene pairs, some *cis*-elements have conserved sequences but divergent chromatin states between rhesus monkeys and humans (Fig. 2a Group II). In Group II, some orthologous genes show similar expression patterns but are regulated by different regulatory elements between rhesus and human PFCs (*n* = 256 orthologous genes, which are regulated by 935 rhesus *cis*-elements or 2800 human *cis*-elements), which is exemplified by the *CALN1* gene (Supplementary Fig. 9c). Our data also show that some orthologous genes with species-specific expression are regulated by species-specific open DHSs. There are 306 orthologous genes with rhesus-specific expression regulated by 1198 rhesus specifically open DHSs, and 742 orthologous genes with human-specific expression regulated by 8408 human specifically open DHSs. For example, *MT1M*, whose regulatory element is specifically open in rhesus monkeys, is specifically expressed in rhesus monkeys but not humans (Supplementary Fig. 10a). To explore why the orthologous genes in Group II are not regulated by the orthologous *cis*-elements between rhesus and human, we have investigated the distances from DHSs to their target genes in one species versus the orthologous regions of DHSs to the corresponding 1-to-1 orthologous genes in the other species. Our data show that the distances between the orthologous regions and the corresponding orthologous genes are comparable between the two species for the conserved regulatory element-gene pairs (Supplementary Fig. 10b). However, the distances are extremely different between the two species for the divergent regulatory element-gene pairs (Supplementary Fig. 10c, d). This suggests that the orthologous DHSs do not regulate the orthologous genes between rhesus monkey and human because of the genome rearrangement. In summary, even though the sequences are conserved, the potential roles of these *cis*-elements in gene expression regulation may be different between rhesus monkey and human. This suggests that we cannot use rhesus monkey as a model to validate the role of these elements in human.

Moreover, our data also show that some regulatory elements of the orthologous genes have species-specific sequences (Group III, *n* = 806 orthologous genes). In this group, the orthologous genes can have conserved (*n* = 114 genes regulated by 202 *cis*-elements with rhesus-specific sequences or 726 *cis*-elements with human-specific sequences) or divergent (*n* = 152 genes with rhesus specific expression regulated by 592 *cis*-elements, *n* = 540 genes with human specific expression regulated by 1983 *cis*-elements) expression patterns between rhesus monkey and human. For example, *CBLN2*, which is linked to the regulatory elements with species-specific sequences in human and rhesus monkey, is mainly expressed before GW16 in human and at E50 stage in rhesus (Supplementary Fig. 10e). *TRADD* is specifically expressed in rhesus monkey and is potentially regulated by *cis*-element with rhesus specific sequence (Fig. 2e).

### *Cis*-element regulates the expression of *BOC* and neuron development

**Fig. 3:**
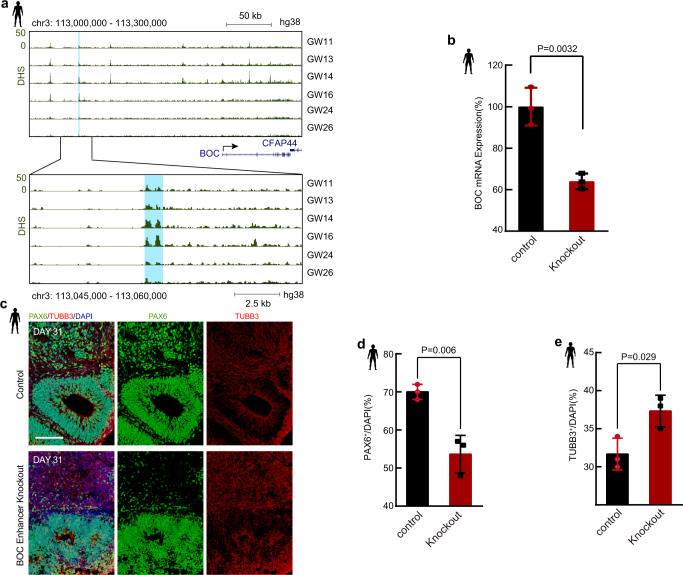
Function of the paired regulatory element on *BOC* in human cortical organoids. **a** Genome browser view of the chromatin accessibility signal around the *BOC* gene during human PFC development. The black arrow indicates the direction of *BOC* transcription. The light blue shadow marks an upstream enhancer of *BOC*. **b** The expression levels of *BOC* in *BOC* enhancer knockout and control human cortical organoids. Student’s *t* test with a two-sided model was used for statistical analysis. *n* = 3 independent replicates. The error bars were defined as the mean values ± SD (standard deviation). **c** Immunostaining for PAX6/TUBB3/DAPI in human cortical organoids at developmental day 31. Scale bar, 50 μm. Representative images are shown from *n* = 3 independent replicates. **d** The percentage of PAX6 + neuron progenitor cells in control and *BOC* enhancer knockout human cortical organoids. Student’s *t* test with a two-sided model was used for statistical analysis. *n* = 3 independent replicates. The error bars were defined as the mean values ± SD. **e** The percentage of TUBB3 + mature neurons in control and BOC enhancer knockout human cortical organoids. Student’s *t* test with a two-sided model was used for statistical analysis. *n* = 3 independent replicates. The error bars were defined as the mean values ± SD.

To explore the regulatory element-gene pairs that are important for PFC development, we first focused on the orthologous genes that harbor conserved DHSs between rhesus monkey and human (Fig. 2a Group I). BOC plays a key role in regulating neuronal differentiation. The depletion of BOC impairs neuronal differentiation^40^. Our data show that *BOC* is mainly expressed before GW16 (Supplementary Fig. 11a). There is a paired regulatory element located approximately 160 kbp upstream of *BOC* (Fig. 3a). The DHS signal of this regulatory element is correlated with *BOC* expression during PFC development (cor =0.92, *p* = 0.0086). To determine whether this regulatory element regulates the expression of *BOC*, we knocked out this regulatory element in human cortical organoids (Supplementary Fig. 11b) by using two single guide RNAs (sgRNAs). Notably, the expression level of *BOC* is significantly decreased in the *cis*-element knockout group (KO) compared to the control group (Fig. 3b), suggesting that the distal element regulates *BOC* expression. To further evaluate the functions of this *cis*-element in neuronal differentiation, we carried out the immunofluorescence staining experiments to assess the cellular composition in cortical organoids by detecting the signals of the neuron progenitor cell marker PAX6, and mature neuron marker TUBB (Fig. 3c). Our data show that the percentage of neuron progenitor cells is significantly decreased in the KO group compared to the control group (Fig. 3d), while the percentage of mature neuron cells is significantly increased in the KO group (Fig. 3e). Collectively, this *cis*-element can regulate *BOC* expression, which is essential for the maintenance of neuron progenitor cells.

### Newly emerged regulatory elements in humans are evolutionarily unstable

**Fig. 4:**
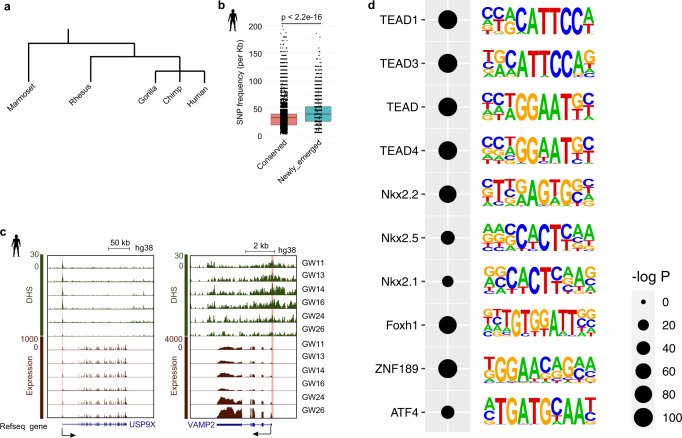
Identification of human newly emerged regulatory elements. **a** The evolutionary tree of primates. **b** Box and jitter plots comparing SNP frequencies (SNP numbers per kb) between newly emerged DHSs (*n* = 3831) and conserved DHSs (*n* = 212,304) in the human population. Boxes and whiskers represent the 25th/75th percentiles and 1.5 $\times$ the interquartile range, respectively. The center in the boxplot represents the medium value. Student’s t test with a two-sided model was used. **c** Genome browser view showing promoter DHS and expression of *USP9X* and *VAMP2* during human PFC development. The pink shadow marks the newly emerged DHSs. **d** Enriched transcription factor binding motifs in newly emerged human DHSs. *P* values are calculated based on the hypergeometric distribution.

During evolution, primate genomes may gain or lose some regulatory elements. As a result, some regulatory elements have newly emerged during human evolution, and some elements are lost in rhesus monkeys but still exist in humans. To investigate the potential effect of evolution on the human PFC, we focused on *cis*-elements that newly emerged in humans but not rhesus monkeys. By comparing the genomic sequences of marmosets, gorillas and chimpanzees, we identified 3831 newly emerged regulatory elements in humans (Fig. 4a). Then, we calculated the frequencies of single nucleotide polymorphism sites (SNPs) discovered in 1000 Genomes Project^41^ in these regulatory elements (see Methods). Our data show that the SNP frequencies in these newly emerged *cis*-elements are significantly higher than those in the conserved *cis*-elements between rhesus and human PFC (Fig. 4b), suggesting that these newly emerged regulatory elements are evolutionarily unstable and have a high mutation risk.

Then we explored the distribution of these newly emerged regulatory elements in the human genome. Our data show that 23% of the newly emerged regulatory elements are located in the promoter region. For example, USP9X and VAMP2 play critical roles in intellectual disability and autism diseases, respectively^42,43^. Both of the genes harbor newly emerged regulatory elements in their promoters. In addition, they are expressed during human PFC development (Fig. 4c).

We then performed TF binding motif enrichment analysis for DHSs that are newly emerged in humans. The results show that the binding motifs of TEAD1, TEAD3, TEAD4 and NKX2 are enriched in these DHSs (Fig. 4d). The TEAD family plays key roles in the Hippo signaling pathway which is involved in the control of organ size^44^. Moreover, NKX2 plays crucial roles in brain diseases^45,46^. These data may provide clues for understanding the differences in brain size between rhesus monkeys and humans and help to explain the occurrence of human neurological diseases.

### Genetic features of species-specific DHSs

**Fig. 5:**
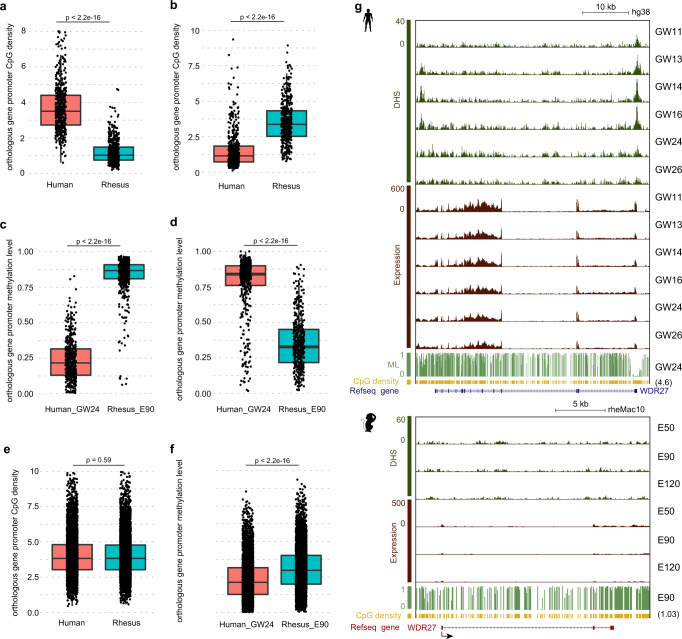
The association between CpG densities and species-specific DHSs. **a** Box and jitter plots comparing promoter CpG densities of orthologous genes, whose promoters are specifically open in the human PFC (*n* = 470 genes). Boxes and whiskers represent the 25th/75th percentiles and 1.5 $\times$ the interquartile range, respectively. The center in the boxplot represents the medium value. Student’s *t* test with a two-sided model was used in this figure. **b** Box and jitter plots comparing promoter CpG densities of orthologous genes, whose promoters are specifically open in rhesus PFC (*n* = 430 genes). Boxes and whiskers represent the 25th/75th percentiles and 1.5 $\times$ the interquartile range, respectively. The center in the boxplot represents the medium value. Student’s *t* test with a two-sided model was used in this figure. **c** Box plots showing DNA methylation levels of orthologous genes, whose promoters are specifically open in humans (*n* = 470 genes), at the human GW24 stage and the E90 stage in rhesus monkeys, respectively. Boxes and whiskers represent the 25th/75th percentiles and 1.5 $\times$ the interquartile range, respectively. The center in the boxplot represents medium value. Student’s *t* test with a two-sided model was used in this figure. **d** Box plots showing DNA methylation levels of orthologous genes, whose promoters are specifically open in rhesus (*n* = 430 genes), at rhesus E90 stage and human GW24 stage, respectively. Boxes and whiskers represent the 25th/75th percentiles and 1.5 $\times$ the interquartile range, respectively. The center in the boxplot represents the medium value. Student’s *t* test with two-sided model was used in this figure. **e** Box plots comparing promoter CpG densities of orthologous genes whose promoters are open in both human and rhesus (*n* = 6683 genes) PFC. Boxes and whiskers represent the 25th/75th percentiles and 1.5 $\times$ the interquartile range, respectively. The center in the boxplot represents the medium value. Student’s *t* test with a two-sided model was used in this figure. **f** Box plots showing DNA methylation levels of orthologous genes whose promoters are conserved open in humans and rhesus monkeys (*n* = 6683 genes), at human GW24 stage and rhesus E90 stage, respectively. Boxes and whiskers represent the 25th/75th percentiles and 1.5 $\times$ the interquartile range, respectively. The center in the boxplot represents the medium value. Student’s *t* test with a two-sided model was used in this figure. **g** Genome browser view of promoter DHS, RNA expression, DNA methylation level (ML) and CpG density of *WDR27* in humans (top) and rhesus monkeys (bottom). The number in parenthesis indicates the promoter CpG density per 100 bp.

The epigenetic states of many promoters are dramatically different in rhesus monkeys and humans. To determine the link between genetic sequence and chromatin accessibility landscape, we calculated promoter CpG densities of orthologous genes between rhesus monkeys and humans. Our data show that orthologous gene promoters that are specifically open in humans show higher CpG densities in humans than in rhesus monkeys (Fig. 5a). Likewise, orthologous gene promoters that are specifically open in the rhesus PFC also show higher CpG densities in rhesus monkeys than in humans (Fig. 5b). Previous studies have shown that CpG densities are anticorrelated with DNA methylation levels^47,48^. In this regard, we also checked DNA methylation states for those species-specific open promoters. As expected, DNA methylation levels of promoters that are specifically open in the human PFC are lower in humans than rhesus monkeys (Fig. 5c). A similar result is observed in the rhesus specifically open promoters (Fig. 5d). We also calculated CpG densities of promoters that are open in both rhesus monkeys and humans, showing that CpG densities of these orthologous promoters are comparable between rhesus monkeys and humans (Fig. 5e). The methylation levels of these promoters show slight differences between rhesus monkeys and humans (Fig. 5f), but the difference is much less than that in Fig. 5c, d. For example, *WDR27*, which is associated with insomnia symptoms^49^, has a high CpG promoter that is open and hypomethylated in humans but not rhesus monkeys (Fig. 5g). Gene expression data also show that *WDR27* is expressed in human but not rhesus PFC. The promoter of *GCSAML*, which is specifically open in rhesus, has higher CpG density, hypomethylated state and higher expression level in rhesus monkeys than in humans (Supplementary Fig. 12). Furthermore, we wondered whether those genes whose promoters were specifically open in humans  are specifically expressed in the brain. Thus, we analyzed the expression patterns of these genes in various tissues. The results show that 265 genes are broadly expressed while only 58 genes show brain-specific expression. This suggests that the phenomenon, that orthologous gene promoters with species-specific DHSs show higher CpG densities in the corresponding species, might not be limited to the PFC. In summary, the evolution of promoter epigenetics in the PFC is linked to CpG density.

### The relationship between human educational attainment or cognitive performance and chromatin accessibility during PFC development

**Fig. 6:**
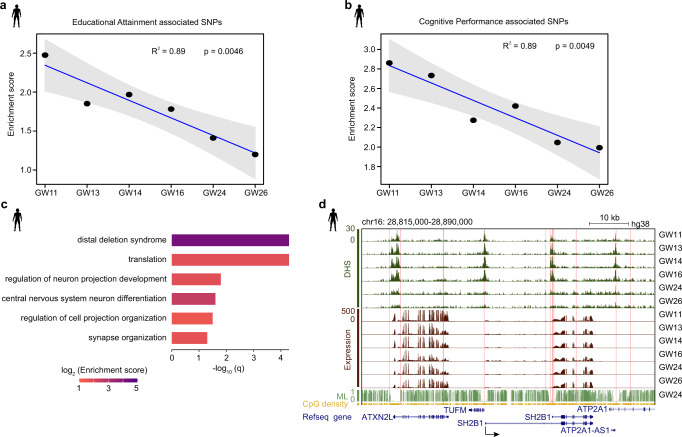
The relationship between human cognitive performance and chromatin accessibility during PFC development. **a** Enrichment of educational attainment associated SNPs (*n* = 2191) in DHS regions in human PFCs exhibits a decreasing trend during development. Gray shading indicates 95% confidence intervals. **b** Enrichment of cognitive performance associated SNPs (*n* = 323) in DHS regions in human PFCs exhibits a decreasing trend during development. Gray shading indicates 95% confidence intervals. **c** GO analyses for educational attainment and cognitive performance SNP associated genes. *P* values are calculated based on the accumulative hypergeometric distribution and q-values are the multiple test adjusted *p* values and multiple testing is performed with the Banjamini-Hochberg method. **d** Genome browser view of the DHS signal near *SH2B1* and RNA expression of *SH2B1*. Pink shadows mark the locations of SNPs which are associated with educational attainment and cognitive performance.

The PFC functions in memory, learning and many different intelligent processes^50,51^. Here, we were interested in the relationship between human intelligence and chromatin accessibility. Educational attainment and cognitive performance are two indicators of the human intelligence level. A previous genome-wide association study (GWAS) identified SNPs that are associated with educational attainment and cognitive performance^52^. Here, we performed enrichment analysis of these SNPs in DHS regions during human PFC development. The data show that educational attainment-associated SNPs are highly enriched in DHS regions of the human PFC, and the enrichment level exhibits a decreasing trend during PFC development (Fig. 6a, Supplementary Fig. 13a). A similar decreasing trend is observed for cognitive performance-associated SNPs (Fig. 6b, Supplementary Fig. 13b). Additionally, we performed the enrichment analyses in human-rhesus conserved and human-specific DHSs, respectively. Our data show that both educational attainment-associated SNPs and cognitive performance-associated SNPs are highly enriched in human-rhesus conserved DHS regions in the human PFC (Supplementary Fig. 13c, d), although there is no decreasing trend of the enrichment during PFC development. In contrast, neither of these two kinds of SNPs are enriched in human-specific DHS regions. Then, we performed GO analysis on the educational attainment and cognitive performance-linked genes whose promoters or distal regulatory elements have both DHSs and SNPs (see Methods). Our data demonstrate that these genes are significantly enriched in synapse organization (Fig. 6c). This is consistent with the fact that synapses play important functions in learning and memory^39^. For example, Src-homology-2 B adaptor protein-1 (SH2B1) is indispensable for brain growth, and SH2B1 mutations in humans are linked to aberrant behavior^53^. Our data show that many SNPs associated with educational attainment or cognitive performance are located around *SH2B1* gene (Fig. 6d). Acylaminoacyl-peptide hydrolase (APEH) has been reported to be associated with Seckel syndrome and Alzheimer’s disease (AD)^54,55^. Our data indicate that *APEH* has an open promoter and is expressed during human PFC development. A SNP located in the promoter of *APEH* is highly associated with both cognitive performance and educational attainment (Supplementary Fig. 13e). These results suggest that chromatin accessibility patterns at the early fetal stage may be important for human educational attainment and cognitive performance.

## Discussion

Recently, significant advances have been achieved in studying PFC development by using high throughput RNA and epigenetic sequencing^31,56–60^. Evolutionary comparison analyses of RNA expression show that the overall expression pattern is conserved between rhesus monkeys and humans^26,58,59^. Our expression data are consistent with previous data. However, we have identified a large number of divergent regulatory element-gene pairs during rhesus and human PFC development. Genes in these divergent regulatory element-gene pairs are highly enriched in the regulation of neuronal synaptic plasticity which plays key roles in cognitive capacity. Moreover, the de novo motif analysis of newly emerged DHSs in the human genome also shows that there are cognitive-associated TFs enriched in these new DHSs. Taken together, our data suggest that the divergence of *cis*-elements may contribute to the difference in cognitive capacity between rhesus monkeys and humans.

On the other hand, most of the expressed genes with promoters harboring the newly emerged DHSs during human PFC development are linked to human neurological diseases, such as schizophrenia and autism. The SNP frequencies of these newly emerged DHSs are significantly higher than the SNP frequencies of conserved DHSs. Perhaps these *cis*-regulatory elements have not been very well evolved during the short evolutionary period from non-human primates to modern humans. The evolutionary selection of these *cis*-elements is still ongoing. Some of the mutations in the *cis*-elements may lead to improper regulation of gene expression, which may cause neurological diseases.

Taken together, our data provide an evolutionary development view for comprehensive understanding of the conservation and divergence in PFC development between humans and non-human primates. Our data are also a valuable resource for the future research on early PFC development in humans and non-human primates.

## Methods

### Ethics of human project

The deidentified human tissue collection and research protocols were approved by the Reproductive Study Ethics Committee of Beijing Anzhen Hospital (HW20131104), the institutional review board (ethics committee) of the Institute of Biophysics and the Ethics Committee of the Beijing Institute of Genomics (2109S003).

Patients with discontinued pregnancies due to complications (such as cervical insufficiency, heart disease, inevitable abortion, eclampsia, etc.) were recruited for this study Fetal tissue samples were collected with signed informed consent from donor patients. Donor ages were not obtained due to privacy protection. The sexes of human embryos were not determined. No compensation was offered for the donors. The collection and other experiments of human tissues in this study were carried out according to the Human Biomedical Research Ethics Guidelines (set by National Health Commission of the People’s Republic of China on Dec. 1st, 2016), the 2016 Guidelines for Stem Cell Research and Clinical Translation (issued by the International Society for Stem Cell Research, ISSCR) and the Human Embryonic Stem Cell Research Ethics Guidelines (set by China National Center for Biotechnology Development on Dec. 24th, 2003). All the protocols are in compliance with the Interim Measures for the Administration of Human Genetic Resources, administered by the Ministry of Science and Technology of China. No statistical methods were used to predetermine the sample size.

### Ethics of animal experiments

All monkey sample collection and other experimental procedures were approved by the Ethics Committee of the Institute of Zoology and Kunming Institute of Zoology, Chinese Academy of Sciences (IACUC18027) and the Ethics Committee of the Beijing Institute of Genomics (2019A019). All the experiments in this study are in compliance with these relevant ethical regulations. No statistical methods were used to predetermine the sample size.

### Human PFC collection

Fetal brains were collected in ice-cold artificial cerebrospinal fluid containing 125.0 mM NaCl, 26.0 mM NaHCO_3_, 2.5 mM KCl, 2.0 mM CaCl_2_, 1.0 mM MgCl_2_, and 1.25 mM NaH_2_PO_4_ at pH 7.4 under oxygenated conditions (95% O_2_ and 5% CO_2_). The tissues of the prefrontal cortex were dissected into small pieces in hibernate E medium (Invitrogen, A1247601) and then stored in liquid nitrogen, which were used for subsequent sequencing library construction.

### Rhesus monkey PFC collection

The study of rhesus monkey embryos at embryonic day 50 (E50) and E90 was approved by the Kunming Primate Research Center, Kunming Institute of Zoology Chinese Academy of Sciences, Kunming, China. The rhesus embryos at E120 were obtained from Hengshu Bio-Technology Company, Sichuan, China. For each embryonic stage, two embryos were collected. The fetal monkeys were obtained from cesarean surgery. Then they were killed by euthanasia. The sexes of the rhesus embryos were not determined. The tissues of the prefrontal cortex were dissected into small pieces and stored in liquid nitrogen, which were used for subsequent sequencing library construction.

### DNase-seq

DNase-seq experiments were carried out as described with some modifications^28,29^. Briefly, the frozen tissue was incubated in a water bath at 37 °C for 10 seconds to be unfrozen and then cut into small pieces on ice using a scalpel. The dissected tissue was resuspended in 200 µL 0.1% BSA/PBS and then transferred into 1.7 mL tubes. After centrifugation at 800 rpm for 5 min at 4 °C, the supernatant was removed. The pellet was resuspended with 180 µL cold lysis buffer (10 mM Tris-HCl pH 7.5, 10 mM NaCl, 3 mM MgCl_2_, 0.1% Triton X-100) by gentle pipetting, and then kept on ice for 30 min. Then, 20 µL diluted DNase I (Roche, 04716728001) was added to final concentration of 80 U/mL and incubated at 37 °C for exactly 5 min. The reaction was stopped by adding 400 µL of stop buffer (10 mM Tris-HCl pH 7.5, 10 mM NaCl, 0.15% SDS, 10 mM EDTA) containing 200 µg Proteinase K (QIAGEN, 19133) followed by incubation at 55 °C for 3 h. The DNA was extracted by phenol-chloroform (Amresco, 0883) and precipitated by ethanol with 20 µg glycogen (Thermo Fisher, R0551) and 1/10 volume 3 M NaOAc (Thermo Fisher, R1181) at −80 °C overnight. Following centrifugation at 12,000 × *g* for 15 min, the DNA precipitate was washed with 800 µL ice-cold 70% ethanol and then dissolved in 50 µL TE (2.5 mM Tris-HCl pH 7.5, 0.05 mM EDTA) after air-drying. The DNA was run on a 2% agarose gel and the DNA band between 50 bp and 100 bp was cut from the gel, followed by purification using a Zymoclean Gel DNA Recovery Kit (ZYMO RESEARCH, D4008). An NEBNext Ultra II DNA Library Prep Kit for Illumina (NEB, E7645S) was used to construct the library. DNA was end repaired and A-tailed by adding 7 µL NEBNext Ultra II End Prep Reaction Buffer and 3 µL NEBNext Ultra II End Prep Enzyme Mix. Samples were incubated in a thermal cycler at 20 °C for 30 min, 65 °C for 30 min, and finally cooled to 4 °C. Adaptor ligation was performed by adding 30 µL NEBNext Ultra II Ligation Master Mix, 1 µL NEBNext Ligation Enhancer, 0.5 µL 200 mM ATP and 2.5 µL Y-shaped Illumina Multiplexing Adaptors (15 µM). Samples were thoroughly mixed and incubated at 20 °C for 30 min. After adaptor ligation, 1.3 volumes of SPRIselect beads (Beckman Coulter, B23318) were used to purify DNA and 10-13 cycles of PCR amplification were performed with NEBNext Ultra II Q5 Master Mix. The PCR product was purified with 1.3 volumes of SPRIselect beads. The libraries were sequenced on a Hiseq X10 with 150 bp paired-end sequencing (Illumina).

### Total RNA-seq library generation

RNA was extracted using a Quick-RNA MicroPrep Kit (ZYMO RESEARCH, R1050) according to the manufacturer’s instructions. Then, ribosomal RNA was removed using an NEBNext rRNA Depletion Kit (NEB, E6310). An NEBNext Ultra II Directional RNA Library Prep Kit for Illumina (NEB, E7765s) was used to construct library according to the manufacturer’s instructions. The libraries were sequenced on a Hiseq X10 with 150 bp paired-end sequencing (Illumina).

### Knockout of target gene enhancer

We designed sgRNAs targeting the putative BOC enhancer by using the GPP sgRNA Designer (CRISPRko) (https://portals.broadinstitute.org) and then optimized sgRNA sequences to maximize the activity and minimize the off-target effects of CRISPR-Cas9. The sgRNA sequences were as follows: BOC sgRNA1: GCC ATA ACG ACT GAT CTC TG, BOC sgRNA2: AAT TAG AAA CAG GCG CCA TG.

The sgRNAs were cloned into the HP180-CBH-Cas9-CMV-EGFP or HP180-CBH-Cas9-CMV-RFP plasmids. The human H9 embryonic stem cells used in this study were purchased from ATCC. They were tested negative for mycoplasma contamination. In addition, the H9 embryonic stem cells were validated by their morphology, gene expression patterns and organoid formation ability. Human H9 embryonic stem cells were transfected with the above plasmids by electroporation using a Lonza AMAXA 4D-Nucleofector. The transfected human H9 embryonic stem cells were cultured for two days. Then the GFP and RFP double positive cells were isolated into a 96-well plate coated with Matrigel by fluorescence-activated cell sorting (FACS) (one cell per well). The genomic DNA of each clone was extracted to examine the BOC enhancer knockout effect by PCR using the following primers: BOC forward primer: GAA GGC ATA AGA CTA ATA CG; reverse primer: AGT GAA ATG ACT TGA CCC. The BOC enhancer knockout clones were further validated by sequencing.

### qRT-PCR of target genes

Total RNA was isolated from enhancer knockout H9 embryonic stem cells using the SV Total RNA Isolation System (Z3100, Roche). RNA concentration was measured with a Nanodrop and RNA integrity was validated by agarose gel electrophoresis. cDNA was prepared using the PrimeScriptTM II1st Strand cDNA Synthesis Kit (6210, TaKaRa). qRT-PCR was performed on a PCR biosystems QuantStudio 7 Flex instrument (Applied Biosystems) with FS Universal SYBR Green Master (4913914001, Roche). We used the following qRT-PCR primers: BOC forward primer: ACG GCG TGG AGA GGA ATG A, reverse primer: GAG GGA CCT CGT TCA AGT CAG. GAPDH (endogenous control) forward primer: CCA TGG GTG GAA TCA TAT TGG A, reverse primer: TCA ACG GA TTT GGT CGT ATT GG. The expression level of target genes was normalized to GAPDH and analyzed using ΔΔCT. The experiment was repeated three times independently.

### Generation and immunostaining of human cortical organoids

Human H9 embryonic stem cells were maintained in Essential 8^TM^ Medium (A1517001, Gibco) on 6-well plates coated with Matrigel (354277, Corning). On day 0, we dissociated the target cell colonies into single cells with Accutase (A1110501, Gibco) and suspended them in 100 cells/μL in KSR medium: DMEM/F-12 (11320082, Gibco), 20% KSR (A3181502, Gibco), 2 mM GlutaMax-I (35050061, Gibco), 0.1 mM NEAA (11140076, Gibco), 0.1 mM beta-mercaptoethanol (21985023, Gibco) with freshly added 10 μM SB431542(1614,TOCRIS), 0.1 μM LDN-193189 (6032,TOCRIS), 3 μM endo-IWR1 (3532,TOCRIS). Then we transferred the cells to 96-well V-bottom plates. We replaced half of the medium with fresh medium once every day until day 18. On day 18, the medium was replaced with neural induction medium containing DMEM/F12, 1:100 N2 supplement (17502048, Gibco), 2 mM GlutaMax-I, 0.1 mM NEAA, and 0.1 μM beta-mercaptoethanol, and organoids were transferred to 24-well low-cell-adhesion plates. Half of the medium was replaced on alternate days.

Organoids were fixed with 4% paraformaldehyde in PBS for 1 h at 4 °C, cryoprotected in 30% sucrose and embedded in optimal cutting temperature medium. Cryosections (25 μm) were collected on Superfrost slides using a Leica CM3050S cryostat. The slices were blocked with 10% donkey serum in PBS with 0.1% Triton X-100 at room temperature for 1 h, and then incubated with rabbit anti-PAX6 (901301, BioLegend, 1:200 dilution with blocking buffer) and mouse anti-TUBB3 (801201, BioLegend, 1:200 dilution with blocking buffer) primary antibodies at 4 °C overnight. After washing three times for 5 min each with washing buffer (0.1% Triton X-100 in PBS) at room temperature, the slides were incubated with Alexa Fluor 488 conjugated donkey anti-rabbit (ab150073, Abcam, 1:200 dilution with blocking buffer) and Alexa Fluor 594 conjugated donkey anti-mouse secondary antibodies (ab150108, Abcam, 1:200 dilution with blocking buffer) at room temperature for 1 h. After washing five times for 5 min each with washing buffer (0.1% Triton X-100 in PBS) at room temperature, the slides were mounted with ProLong Gold Antifade Reagent with DAPI (8961 S, Cell Signaling Technology) for imaging. Images were collected using an Olympus FV3000 confocal microscope.

### Whole genome bisulfite sequencing (WGBS)

WGBS experiments were carried out as described^61^. Briefly, DNA was extracted using a QIAamp DNA Mini Kit (QIAGEN, 51306). Then, 0.1% unmethylated lambda DNA was added to the DNA sample. Then they were sonicated to 300–500 bp by using Covaris S2 (Applied Biosystems). End-repair, dA-tailing and methylated adaptor ligation were performed using the NEBNext Ultra II DNA Library Prep Kit for Illumina (NEB, E7645S). After adaptor ligation, 1.3 volumes of SPRIselect beads (Beckman Coulter, B23318) were used to purify DNA. Bisulfite conversion was performed using the EZ DNA methylation-Gold Kit (Zymo Research, D5005) according to the manufacturer’s instructions. Bisulfite-treated DNA was amplified using KAPA HIFI HotStart Uracil+ ReadyMix (KAPA, kk2802) with 9–10 cycles. The libraries were sequenced on a Hiseq X10 with 150 bp paired-end sequencing (Illumina).

### DNase-seq data analysis

We first removed the low-quality bases in the reads of DNase-seq data and then cropped the reads to 36 bp by Trimmomatic v0.33^62^. Only read 1 reads were used for mapping. Human libraries were aligned to hg38 and rhesus libraries were aligned to RheMac10 by Bowtie v1.2.0^63^ with the parameter “ -m 1”. Samtools v1.3.1^64^ was used to remove low mapping quality reads (MAPQ < 10) and Picard^65^ was used to remove PCR duplicated reads. To exclude the effect of sequencing depth, we sorted the alignment file by read name for each replicate and then extracted the first 20 million reads for further analysis.

We split the genome into nonoverlapping 2 kb windows and calculated the fragment per kilobase per million mapped reads (FPKM) values for all windows which were used as tag densities of DNase-seq data. The Pearson correlation coefficient of tag densities between two replicates was calculated. Then we merged the reads from two replicates. DHSs were called by hotspot algorithm v4.1 with FDR < 0.01^66^. Only DHSs with P values less than 1e-5 were retained for further analysis. DNase-seq tracks visualized in the UCSC Genome Browser were generated by bamCoverage in Deeptools v2.5.7 suite with the parameter “—normalizeUsing RPKM”^67^.

We overlapped the DHSs with human and rhesus cortex putative enhancers and promoters from ref. ^31^. The DHSs from different developmental stages were combined. Then we extended the human DHSs both upstream and downstream by 500 bp, and merged the DHSs if they are overlapped. Next, we used the UCSC “liftOver” tool to convert the coordinates of the published putative enhancers and promoters in the human hg19 genome to the hg38 genome. The extended DHSs were used to overlap with human putative enhancers and promoters. For rhesus monkeys, the merged DHSs were first mapped to the RheMac8 reference by the UCSC “liftOver” tool. Then, the published putative enhancers and promoters in rhesus monkeys were also mapped to the RheMac8 reference. Next, the DHSs and rhesus cortex putative enhancers and promoters were used to perform overlapping analyses.

### Genomic annotation

The GRCh38 and Macaque (Mmul_10) refGene files from Ensembl were used for genome annotation. We used TSS (transcript start site) $\pm$ 2kb as the promoter region. If a DHS overlapped with a promoter region, the DHS was assigned as a promoter DHS. If a DHS overlapped with the gene body but did not overlap with the promoter, the DHS was assigned as a gene body DHS. The other DHSs were assigned as intergenic DHSs. We used Bedtools v2.26.0^68^ for this analysis.

If a promoter overlaps with a DHS, then the promoter is defined as an open promoter.

Information on orthologous genes between humans and rhesus monkeys was obtained from Ensembl BioMart. The genes that have only one orthologous gene in both species are used for further analysis.

Moreover, human-specific open promoters mean that the orthologous gene promoters are open in each developmental stage of humans but not in any developmental stage of rhesus monkeys. Similarly, rhesus-specific open promoters mean that the orthologous gene promoters are open in each developmental stage of rhesus monkeys but not in any developmental stage of humans. Additionally, the human-rhesus conserved open promoter means that the orthologous gene promoter are open in both humans and rhesus monkeys at each stage.

The CpG number in the promoter was counted by a custom script. Promoter CpG density was defined as the average number of CpG sites per 100 bp.

### RNA-seq data analysis

Low quality bases in reads were trimmed by Trimmomatic with default parameters. For human RNA-seq data, we mapped the paired-end reads to hg38 using HISAT v2.0.4 with the parameter “-dta-cufflinks”^69^. For rhesus data, paired-end reads were aligned to rheMac10 by HISAT v2.0.4 with the parameter “-dta-cufflinks”. We used Cufflinks v2.2.1^70^ to calculate the FPKM value of each gene. The Pearson correlation coefficient of gene expression between two replicates was calculated. Then we merged the alignment files for two replicates and used Cufflinks v2.2.1 to recalculate the FPKM value of each gene, which was used as the expression level of each gene for further analysis. The genome reference files were downloaded from Ensembl. Genes with FPKM values < 1 were considered as genes that were not expressed. RNA-seq tracks visualized in the UCSC Genome Browser were generated by bamCoverage in Deeptools v2.5.7 suite with the parameter “—normalizeUsing RPKM”.

### Identification of dynamically expressed genes

To quantitatively measure the dynamics of gene expression, we calculated the entropy score for each gene by using a previously described method^71^. We first normalized the expression levels (log_2_ FPKM) by the quantile normalization method among different developmental stages. Then we calculated the entropy score for each gene. In humans, we regard the genes with entropy scores < 2.55 as stage-specific expressed genes. In rhesus monkeys, we regard the genes with entropy scores < 1.56 as stage-specific expressed genes.

### The dynamics of gene expression

To investigate the dynamics of gene expression at different stages, we first normalized the expression levels (log_2_ FPKM) by the quantile normalization method among different development stages. Then, for genes that were stage specifically expressed, we normalized the expression levels of those genes at different development stages based on max expression level and min expression level. The equation is:

1 $$\frac{x-{{{\min }}}_{{{{{{\rm{expression}}}}}}\; {{{{{\rm{level}}}}}}}}{{{{\max }}}_{{{{{{\rm{expression}}}}}}\; {{{{{\rm{level}}}}}}}\,-{{{\min }}}_{{{{{{\rm{expression}}}}}}\; {{{{{\rm{level}}}}}}}}$$

In this equation, *x* represents the expression level of a gene at one stage; max_expression level_ represents the max expression level of the gene at all development stages, and min_expression level_ represents the min expression level of the gene at all development stages.

### Identification of conserved and divergent DHSs between humans and rhesus monkeys

The DHSs from different developmental stages were combined and the unique DHSs were retained. We used the UCSC “liftOver” tool to convert the coordinates of DHSs in human genome to rhesus genome (hg38 to rheMac10) and multiple hits for a region were allowed when we ran liftover. The DHSs that lifted over to multiple hits were removed for further analysis. Then, we obtained three categories of DHSs in the human genome: (1) The DHSs in the human genome could be converted to the rhesus genome, and they overlapped with the extended rhesus DHSs after conversion to the rhesus genome. (2) The DHSs in human genome could be converted to the rhesus genome, but they did not overlap with any extended rhesus DHSs after conversion to the rhesus genome. (3) The DHSs in the human genome could not be converted to the rhesus genome. Similarly, we used the “liftOver” tool to convert the coordinates of DHSs in rhesus genome to human genome (rheMac10 to hg38). We also obtained three categories of DHSs in the rhesus genome: (1) The DHSs in the rhesus genome could be converted to the human genome and they overlapped with the extended human DHSs after conversion to the human genome. (2) The DHSs in the rhesus genome could be converted to the human genome but they did not overlap with any extended human DHSs after conversion to the human genome. (3) The DHSs in the rhesus genome could not be converted to the human genome.

For the first categories in both humans and rhesus monkeys, only the sequence conserved DHSs in the human and rhesus genomes that were reciprocally overlapped after genome conversion and satisfied the synteny constraint were kept for further analysis. For example, a human DHS region h_peak_1 was converted to the rhesus genome and overlapped with a rhesus DHS region r_peak_1 and the fraction of overlap is over 0.5. In addition, the rhesus DHS region r_peak_1 was converted to the human genome and overlapped with the human DHS region h_peak_1. Similarly, the fraction of overlap should be over 0.5. In addition, we also performed synteny analysis. The genes around h_peak_1 should be consistent with those around r_peak_1. Then, we considered the h_peak_1 in humans and r_peak_1 in rhesus as a pair of conserved DHSs between humans and rhesus monkeys. These paired DHSs share conserved sequences and similar chromatin accessibility between humans and rhesus monkeys. For the second category in humans and rhesus monkeys, if a DHS in the human genome was open at two or more stages during human PFC development, we defined the DHS as human-specific open DHS. Similarly, if a DHS in the rhesus genome was open at one or more stages during rhesus PFC development, we defined the DHS as rhesus-specific open DHS. These DHSs share conserved sequences between humans and rhesus monkeys but they are species-specific open. For the third category in humans and rhesus monkeys, we defined these nonconserved DHSs as species genome specific DHSs.

### Identification of potential gene regulatory elements

We calculated the FPKM of each DHS region, and the log_2_ FPKM value was defined as the chromatin accessibility signal of the DHS region. Moreover, we normalized the accessibility signal at different development stages by the quantile normalization method. The normalized accessibility signal was used for further analysis. Similarly, we normalized the log transformed expression levels of genes (log_2_ FPKM values) at different development stages by the quantile normalization method. Most distal regulatory elements that regulate gene expression are located within 1000 kilobase pairs both upstream and downstream of transcriptional start sites (TSSs)^72,73^. We defined the 998 kbp region both upstream and downstream of the promoter as the gene distal region. For each gene, there are many large DHSs in the promoter and distal regions, we calculated the Pearson correlation coefficients between the normalized chromatin accessibility signal of each DHS region and normalized expression level of the associated gene by using the cor.test function in R. If the Pearson’s correlation coefficient is over 0.8 and the p value is less than 0.05, we called the DHS as the paired regulatory element for the gene. K-means clustering was performed by the k-means function in R v3.6.1 and the cluster number was determined by the sum squared error (SSE) and the genes were clustered based on RNA-seq.

Additionally, for the genes in cluster 6 in humans and rhesus monkeys (Fig. 1c), we investigated whether these genes were regulated by different sets of *cis*-elements at different developmental stages. In detail, we considered that each of these genes was regulated by two different sets of regulatory elements during development. For example, gene 1 was mainly expressed at the E50 and E120 stages but not at the E90 stage in rhesus PFC. We considered that this gene was regulated by two different sets of *cis*-elements. One set of *cis*-elements regulated the expression of this gene only at the E50 stage, but not at the E90 and E120 stages. The other set of *cis*-elements regulated the expression of this gene only at the E120 stage, but not at the E50 and E90 stages. To remove the signal of one of the stages, we assumed that the DHS signal at this stage was 0, and the RNA expression level at this stage was <1. Then we used the correlation approach to identify the DHSs regulating the expression of the gene at the earliest and latest stages, respectively. Using a similar strategy, we identified different sets of putative regulatory elements for each gene in human cluster 6.

### Conserved and divergent regulatory element-gene pairs between humans and rhesus monkeys

If a conserved potential regulatory element between humans and rhesus monkeys potentially regulated the same orthologous gene in humans and rhesus monkeys, we defined this paired regulatory element and orthologous gene as a conserved regulatory element-gene pair (Fig. 2a group I). The remaining regulatory element-gene pairs were defined as divergent element-gene pairs between humans and rhesus monkeys. Based on the chromatin accessibility states of potential regulatory elements, we split the divergent element-gene pairs into two groups (Fig. 2a group II and group III). In group II, the regulatory elements are from two categories: (1) The DHS shares conserved sequence between human and rhesus, but it is species specifically open. (2) The DHS shares a conserved sequence and is open in both humans and rhesus monkeys. However, the chromatin accessibility signal dynamics are not consistent between humans and rhesus monkeys. In group III, the sequence of the DHS is not conserved between humans and rhesus monkeys.

### Identification of tissue-specific or broadly expressed genes

The expression levels (TPMs) of genes in various human tissues were obtained from the Genotype-Tissue Expression (GTEx) Project, which was supported by the Common Fund of the Office of the Director of the National Institutes of Health, and by NCI, NHGRI, NHLBI, NIDA, NIMH, and NINDS. The TissueEnrich package v1.14.0 was used to identify whether the genes were tissue-specific expressed or broadly expressed.

### Validation of regulatory element-gene pairs by Hi-C data

To validate the regulatory element-gene pairs identified by the correlation approach, we integrated a published interaction profile for enhancers in the human cerebral cortex at the midgestation stages (GW17-GW18 stages) for analysis^18^. Overlap analysis was performed by using Bedtools v2.26.0.

### Human genome newly emerged DHSs and rhesus genome specific DHSs

The DHSs from different developmental stages were combined, and the unique DHSs were retained. Then, we converted the coordinates of DHSs in the human genome to the rhesus genome (RheMac10), marmoset genome (CalJac3), gorilla genome (GorGor6) and chimpanzee genome (PanTro6), respectively. Then, we defined the DHSs that could not be converted to any of these four non-human primate genomes as the newly emerged DHSs in the human genome (Fig. 4).

### SNP frequency analysis

Information on SNPs in the human genome was downloaded from http://ftp.1000genomes.ebi.ac.uk/vol1/ftp/release/20130502/. The SNP frequency per kb for each DHS was calculated by the following equation:

2 $${{{{{\rm{SNP}}}}}}\; {{{{{\rm{frequency}}}}}}=\frac{{{{{{\rm{the}}}}}}\; {{{{{\rm{number}}}}}}\; {{{{{\rm{of}}}}}}\; {{{{{\rm{SNPs}}}}}}\; {{{{{\rm{in}}}}}}\; {{{{{\rm{peak}}}}}}}{{{{{{\rm{peak}}}}}}\; {{{{{\rm{length}}}}}}/1000}$$

### Human newly emerged DHS-associated neurological diseases

We obtained the clinical and functional information of each gene in the GeneCards database https://www.genecards.org/. Moreover, we also obtained autism-associated genes from the Simons Foundation Autism Research Initiative (SFARI) database https://gene.sfari.org/database/human-gene/.

### DNA methylation analysis

The bases with low qualities were trimmed by Trimmomatic with default parameters. The 48,502 bp lambda genome was added to the hg38 reference genome and rheMac10 reference genome as an extra chromosome for the calculation of bisulfite conversion rates. For human methylation data, paired-end reads were mapped against the hg38 reference by Bismark v0.7^74^ with default parameters. For rhesus methylation data, paired-reads were mapped against the rheMac10 reference by Bismark. PCR duplicates were removed by Picard v2.5.0 and the overlapping parts of paired-end reads were trimmed from one read of paired reads by bamUtil v1.0.13^75^. The quantification of methylation levels of CpGs was described in our previous work^61^. The methylation level of each CpG site was calculated by the ratio of the number of methylated Cs to the total number of methylated Cs and unmethylated Cs. A custom script was used to calculate the methylation level of each CpG site. The promoter methylation level was measured as the average DNA methylation level of all CpG sites in the region.

### Motif enrichment analysis

We used Homer software v4.11 to perform motif analysis with default parameters, and we used all the DHSs recovered in this study as the background.

### Gene ontology (GO) analysis

GO analysis was performed by Metascape web tool v3.5 with default parameters^76^. *P* values are calculated based on the accumulative hypergeometric distribution and q-values are the multiple test adjusted *p* values and multiple testing is performed with the Banjamini-Hochberg method. All genes in the genome are used as background for the enrichment analysis. The enrichment score (ES) is calculated by the equation:

3 $${{{{{\rm{ES}}}}}}=\frac{{{{{{\rm{the}}}}}}\; {{{{{\rm{number}}}}}}\; {{{{{\rm{of}}}}}}\; {{{{{\rm{membership}}}}}}\; {{{{{\rm{genes}}}}}}\; {{{{{\rm{in}}}}}}\; {{{{{\rm{input}}}}}}\; {{{{{\rm{genes}}}}}}}{{{{{{\rm{the}}}}}}\; {{{{{\rm{number}}}}}}\; {{{{{\rm{of}}}}}}\; {{{{{\rm{input}}}}}}\; {{{{{\rm{genes}}}}}}}/\frac{{{{{{\rm{the}}}}}}\; {{{{{\rm{number}}}}}}\; {{{{{\rm{of}}}}}}\; {{{{{\rm{total}}}}}}\; {{{{{\rm{membership}}}}}}\; {{{{{\rm{genes}}}}}}}{{{{{{\rm{the}}}}}}\; {{{{{\rm{number}}}}}}\; {{{{{\rm{of}}}}}}\; {{{{{\rm{all}}}}}}\; {{{{{\rm{genes}}}}}}\; {{{{{\rm{in}}}}}}\; {{{{{\rm{the}}}}}}\; {{{{{\rm{genome}}}}}}}$$

### SNP enrichment analysis

SNPs associated with educational attainment or cognitive performance were downloaded from https://www.thessgac.org/data. We explored SNP enrichment in accessible chromatin regions. For SNPs associated with a particular type of nervous system disease, we calculated the enrichment score (ES) by the equation:

4 $${{{{{\rm{ES}}}}}}=\frac{{{{{{\rm{the}}}}}}\; {{{{{\rm{number}}}}}}\; {{{{{\rm{of}}}}}}\; {{{{{\rm{SNPs}}}}}}\; {{{{{\rm{in}}}}}}\; {{{{{\rm{peaks}}}}}}}{{{{{{\rm{sum}}}}}}\; {{{{{\rm{of}}}}}}\; {{{{{\rm{peak}}}}}}\; {{{{{\rm{length}}}}}}}/\frac{{{{{{\rm{the}}}}}}\; {{{{{\rm{number}}}}}}\; {{{{{\rm{of}}}}}}\; {{{{{\rm{total}}}}}}\; {{{{{\rm{SNPs}}}}}}}{{{{{{\rm{whole}}}}}}\; {{{{{\rm{genome}}}}}}\; {{{{{\rm{length}}}}}}}$$

If the enrichment score > = 1.5, then we considered that the SNPs associated with such disease were enriched in the accessible chromatin regions. The R package basicTrendline was used to draw the fitting trend line and add a 95% confidence interval. SNPs associated genes are defined if they meet one of following criteria: 1. Protein coding genes with open promoters are expressed in the human PFC, and the promoter regions harbor educational attainment or cognitive performance associated SNPs. 2. Protein coding genes are expressed in human PFC and the distal regulatory elements of the genes are open and harbor educational attainment or cognitive performance associated SNPs.

### Statistical analysis

The statistical analysis was performed by R v3.6.1. Pearson’s correlation coefficient in Supplementary Fig. 1c, d and Supplementary Fig. 3a, b was calculated by cor.test function with default parameters. The Wilcoxon rank sum test with continuity correction was used for gene expression level comparisons in Supplementary Fig. 3c, d, *** represents *p* < 2.2e–16. Student’s *t* test with a two-sided model was used for BOC expression level comparisons in Fig. 3b and the percentage of cell number comparisons in Fig. 3d, e, SNP frequency comparisons in Fig. 4b, CpG density comparisons and methylation level comparisons in Fig. 5. Accumulative hypergeometric distribution was used to calculate the *p* values in GO analysis, and multiple testing with the Banjamini-Hochberg method was performed to adjust *p* values.

### Reporting summary

Further information on research design is available in the Nature Research Reporting Summary linked to this article.

## Supplementary information

Supplementary Information

Description of Additional Supplementary Files

Supplementary Data 1

Supplementary Data 2

Supplementary Data 3

Reporting Summary

## Data Availability

The datasets generated and analyzed during the current study are available in the Genome Sequence Archive under accession numbers CRA002225 and HRA002180. The ChIP-seq data for human and rhesus forebrain analyzed in this study were downloaded from NCBI Gene Expression Omnibus (GEO) under accession GSE63649. The sequences and genomic annotations of reference genomes for human hg38/GRCh38 and rhesus rheMac10/Mmul_10 were downloaded from Ensembl (hg38 genome sequence [http://ftp.ensembl.org/pub/release-104/fasta/homo_sapiens/dna/Homo_sapiens.GRCh38.dna.primary_assembly.fa.gz], hg38 annotation [http://ftp.ensembl.org/pub/release-104/gtf/homo_sapiens/Homo_sapiens.GRCh38.104.chr.gtf.gz], rheMac10 genome sequence [http://ftp.ensembl.org/pub/release-104/fasta/macaca_mulatta/dna_index/Macaca_mulatta.Mmul_10.dna.toplevel.fa.gz], rheMac10 annotation [http://ftp.ensembl.org/pub/release-104/gtf/macaca_mulatta/Macaca_mulatta.Mmul_10.104.chr.gtf.gz]). The SNPs associated with educational attainment or cognitive performance were downloaded from https://www.thessgac.org/data. Information on SNPs in the human genome was downloaded from http://ftp.1000genomes.ebi.ac.uk/vol1/ftp/release/20130502/. The expression levels of genes in various human tissues were downloaded from GTEx Portal V8 [https://storage.googleapis.com/gtex_analysis_v8/rna_seq_data/GTEx_Analysis_2017-06-05_v8_RNASeQCv1.1.9_gene_tpm.gct.gz]. The interaction profile for enhancers in the human cerebral cortex at the midgestation stages (GW17-GW18 stages) was obtained from the supplementary information files in a published article 18.

## Code availability

The code used in this study is available on request.
