# Supplementary material for: Comparison of chromatin accessibility landscapes during early development of prefrontal cortex between rhesus macaque and human
Source: Nat Commun. 2022 Jul 6;13:3883. doi: 10.1038/s41467-022-31403-3 (PMC9259620; doi:10.1038/s41467-022-31403-3)
Supplement: Supplementary file 1 — Supplementary Information [file 41467_2022_31403_MOESM1_ESM.pdf]

Supplementary Information for Comparison of chromatin accessibility landscapes during early development of prefrontal cortex between rhesus macaque and human

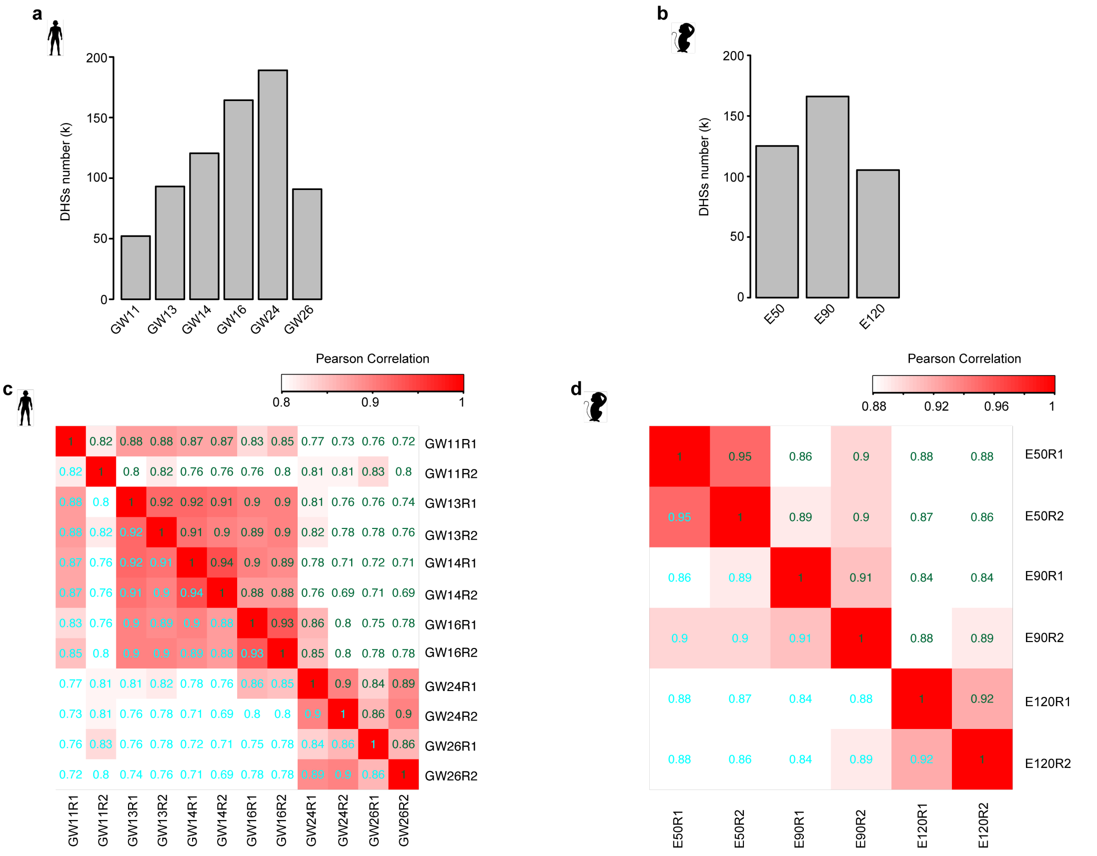

Supplementary Figure 1 | DNase-seq in human and rhesus PFC samples. **a**, Bar plot showing the number of DHSs at each stage in human PFCs during PFC development. **b**, Bar plot showing the number of DHSs at each stage in rhesus PFCs during PFC development. **c**, Heatmap showing Pearson correlation coefficients of DNase-seq tag densities between two replicates for human PFCs. DNase-seq tag densities represent FPKM values of each genome-wide nonoverlapping 2 kb window. FPKM refers to fragment per kilobase per million mapped reads. **d**, Heatmap showing Pearson correlation coefficients of DNase-seq tag densities between two replicates for rhesus PFCs.

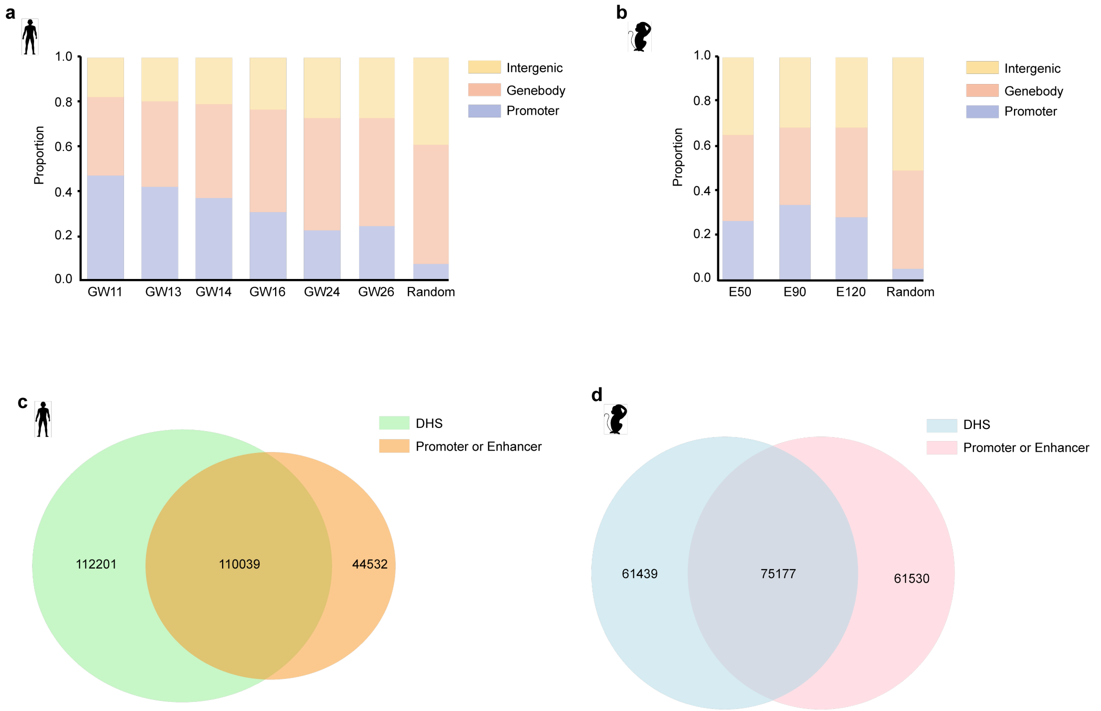

Supplementary Figure 2 | The annotation of DHSs in human and rhesus PFCs. a, Plot showing the genomic distribution of DHSs in human PFCs during PFC development. The distribution of promoter, gene body and intergenic region in the whole genome is referred to as random. **b**, Plot showing the genomic distribution of DHSs in rhesus PFCs during PFC development. **c**, The overlap between human DHSs and active promoters or enhancers identified in a previous study. **d**, The overlap between rhesus DHSs and active promoters or enhancers identified in a previous study.

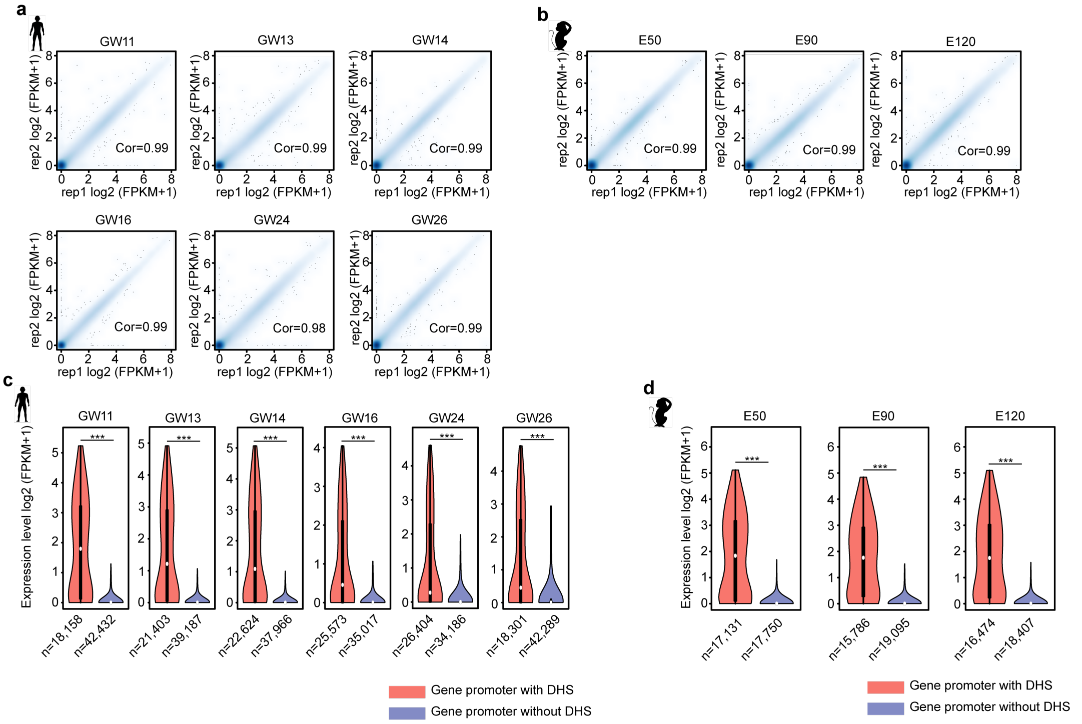

Supplementary Figure 3 | Gene expression in human and rhesus PFCs. **a**, Scatter plots comparing gene expression levels between two replicates for human PFCs at each stage. FPKM values were calculated for all genes. Cor, Pearson correlation coefficients. **b**, Scatter plots comparing gene expression levels between two replicates for rhesus PFCs at each stage. **c**, Violin plots showing the expression levels of genes with or without promoter DHSs in human PFCs at each stage. The Wilcoxon rank sum test with continuity correction was used for statistical analysis. *** represents p value < 2.2e-16. **d**, Violin plots showing the expression levels of the genes with or without promoter DHSs at each stage in rhesus PFCs. The Wilcoxon rank sum test with continuity correction was used for statistical analysis. *** represents p value < 2.2e-16.

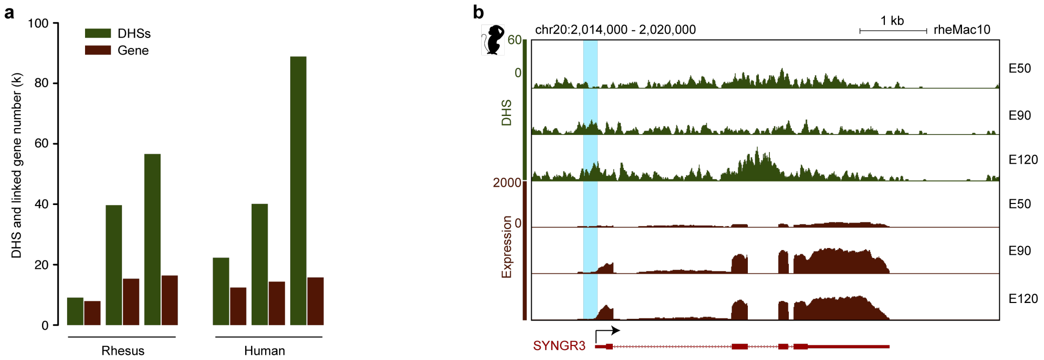

Supplementary Figure 4 | Identification of potential regulatory elements. a, Bar plot shows the numbers of potential cis-regulatory elements in different categories of Fig. 1b and the numbers of the associated genes. **b**, Genome browser view of DNase-seq and RNA-seq signals around *SYNGR3* during rhesus PFC development. The light blue shadows mark the position of a potential regulatory element of *SYNGR3*. The black arrow indicates the direction of *SYNGR3* transcription.

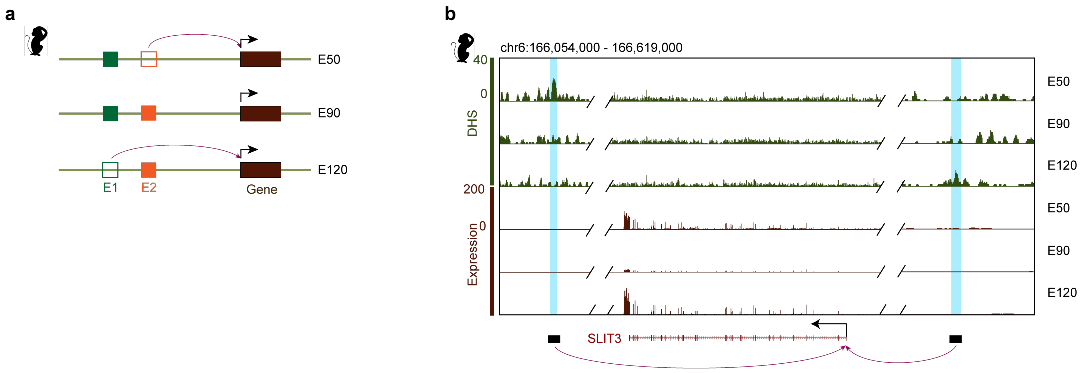

Supplementary Figure 5 | Genes are potentially regulated by different regulatory elements at different developmental stages. **a**, Schematics showing that the gene is associated with different regulatory elements at different developmental stages. E1 is open at the E120 stage but not at the E50 and E90 stages, and E2 is open at the E50 stage but not at the E90 and E120 stages. **b**, Genome browser view of RNA expression of *SLIT3* and the DNase-seq signal around this gene during rhesus PFC development. The light blue shadows mark the positions of two potential regulatory elements of *SLIT3*. The pink arrow indicates the potential regulatory element of this gene. A downstream potential regulatory element (left) is only open at the E50 stage but not at the E90 and E120 stages. However, an upstream potential regulatory element (right) is open at the E120 stage but not at the E50 and E90 stages. The black arrow indicates the direction of *SLIT3* transcription.

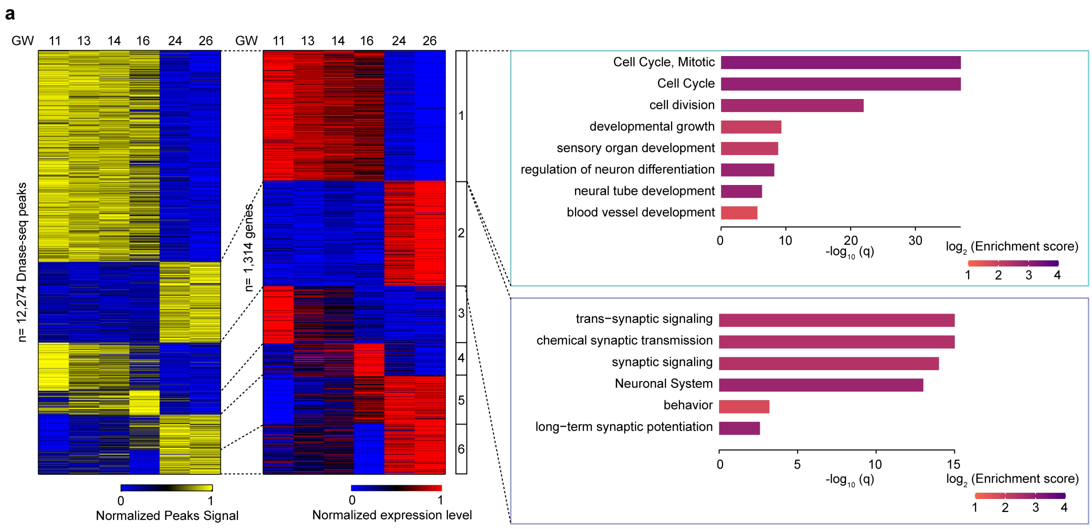

Supplementary Figure 6 | *Cis*-element-gene pairs that were supported by the Hi-C data during human PFC development. a, The dynamics of gene expression and the DHS signal of paired regulatory elements in human PFC, and these *cis*-element-gene pairs are supported by Hi-C data. The regulatory element-gene pairs are classified into 6 clusters by the K-means method. The right panel shows the significantly enriched GO terms in cluster 1 and cluster 2. P values are calculated based on the accumulative hypergeometric distribution, and q-values are the multiple test adjusted p values and multiple testing is performed with the Banjamini-Hochberg method.

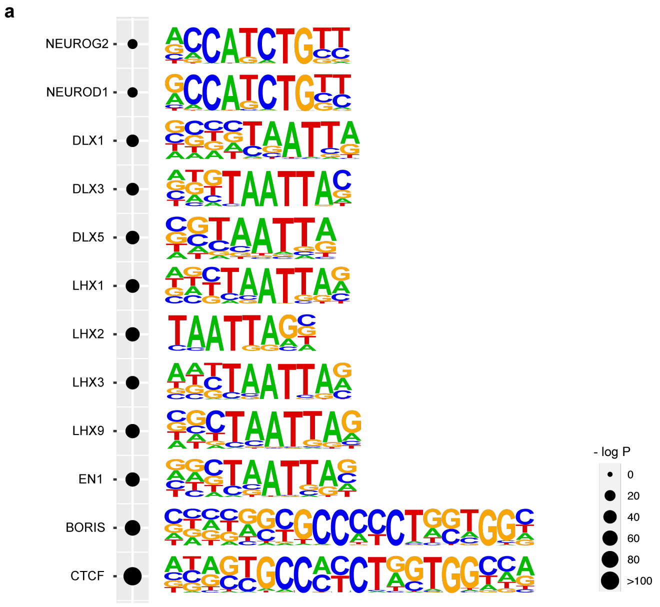

Supplementary Figure 7 | Transcription factor (TF) binding motif enrichment for regulatory elements which are from conserved regulatory element-gene pairs. **a**, Enriched TF binding motifs in regulatory elements which are from conserved regulatory element-gene pairs. The size of the dot for each TF represents the TF binding motif enrichment in regulatory elements. P values are calculated based on the hypergeometric distribution.

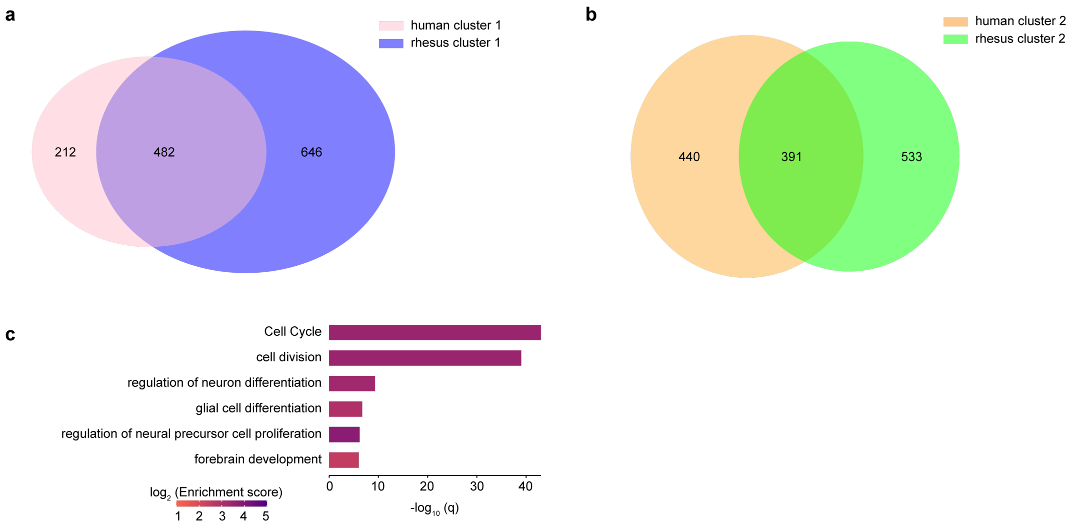

Supplementary Figure 8 | Conserved and divergent expression dynamics of orthologous genes between humans and rhesus monkeys. **a**, The numbers of conserved and species specifically expressed orthologous genes during the early PFC development stage between humans and rhesus monkeys. **b**, The number of conserved and species specifically expressed orthologous genes during the later PFC development stage between humans and rhesus monkeys. **c**, GO enrichment of genes in conserved regulatory element-gene pairs (group 1). P values are calculated based on the accumulative hypergeometric distribution, and q-values are the multiple test adjusted p values and multiple testing is performed with the Banjamini-Hochberg method.

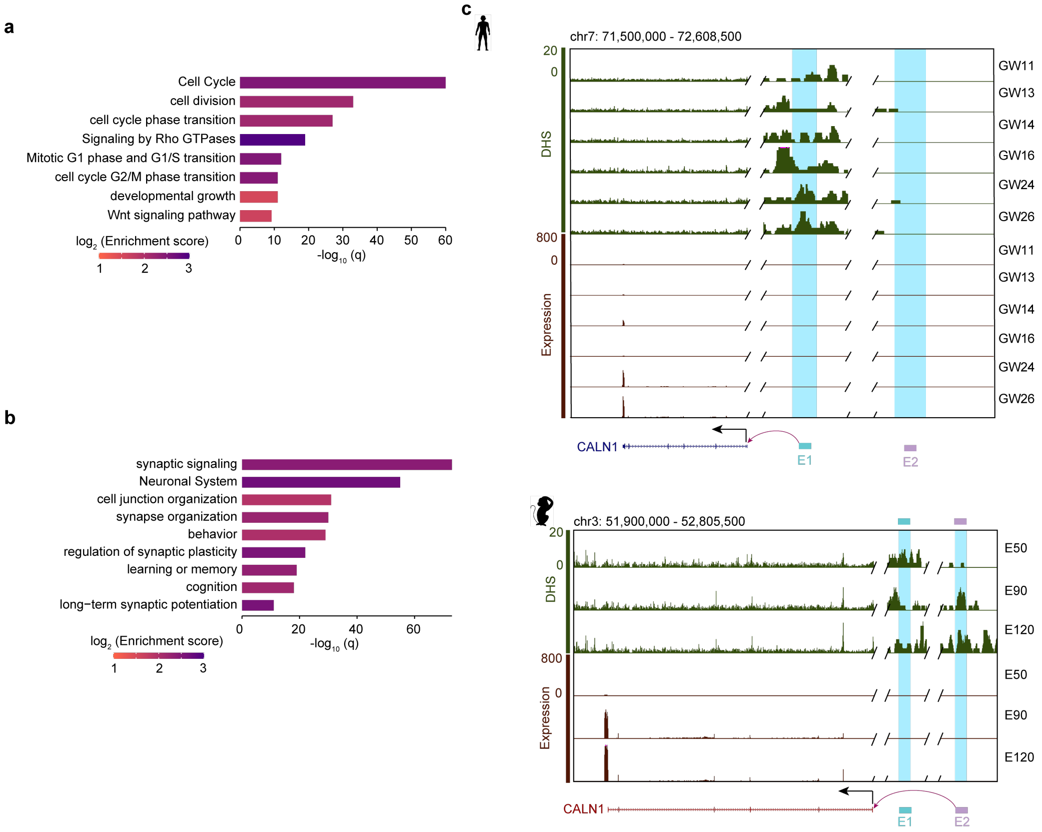

Supplementary Figure 9 | Divergent regulatory element-gene pairs between humans and rhesus monkeys. **a**, GO enrichment of genes in divergent regulatory element-gene pairs that are mainly expressed before GW16 in the human PFC or at the E50 stage in the rhesus PFC. **b**, GO enrichment of genes in divergent regulatory element-gene pairs that are mainly expressed at the GW24 and GW26 stages in human PFC, or at the E90 and E120 stages in rhesus PFC. P values are calculated based on the accumulative hypergeometric distribution. The q-values are the multiple test adjusted p values and multiple testing is performed with the Banjamini-Hochberg method. **c**, Genome browser view of *CALN1* expression and the DHS signal of the paired regulatory elements in humans (top) and rhesus monkeys (rhesus). The light blue shadows mark the paired regulatory elements. Element E1 shares a conserved sequence between humans and rhesus monkeys, but the dynamics of chromatin accessibility of this element are not conserved between humans and rhesus monkeys. E1 is open at the PCW24 and PCW26 stages in human PFC but is open at E50 stage in rhesus PFC. Element E2 shares a conserved sequence, but it is specifically open in rhesus monkeys.

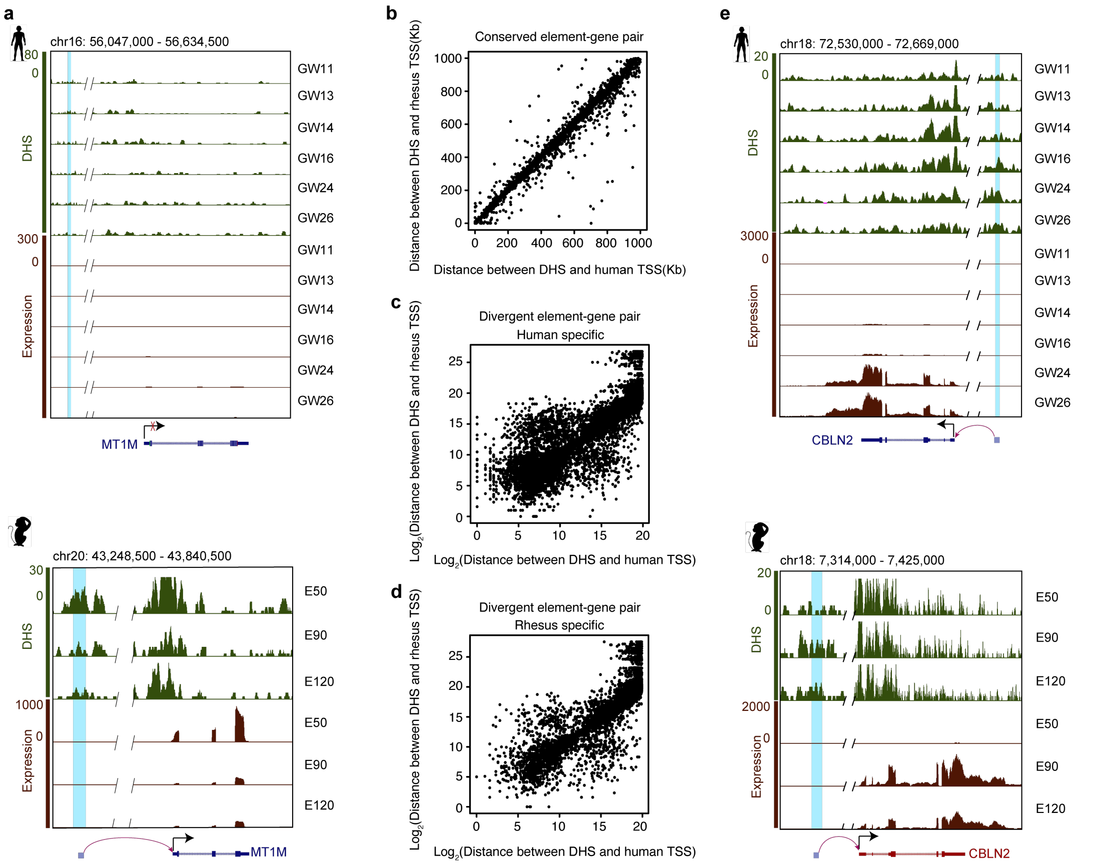

Supplementary Figure 10 | Species-specific chromatin accessibility states of the paired regulatory elements in humans and rhesus monkeys. **a**, Genome browser showing that *MT1M* is specifically expressed in the rhesus PFC (bottom) but not in the human PFC (top). The light blue shadow marks the position of a potential regulatory element of this gene in rhesus monkeys. This element shares a conserved sequence, but it is specifically open in rhesus monkeys. **b,** Scatter plot showing the distances from DHSs to their target genes in humans versus the orthologous regions of DHSs to the corresponding 1-to-1 orthologous genes in rhesus monkeys for the conserved regulatory element-gene pairs. **c,** Scatter plot showing the distances from DHSs to their target genes in humans versus the orthologous regions of DHSs to the corresponding 1-to-1 orthologous genes in rhesus monkeys for human specific *cis*-element-gene pairs. **d,** Scatter plot showing the distances from DHSs to their target genes in rhesus monkeys versus the orthologous regions of DHSs to the corresponding 1-to-1 orthologous genes in humans for rhesus specific *cis*-element-gene pairs. **e,** Genome browser view of *CBLN2* expression and the DHS signal of the paired regulatory element during human (top) and rhesus PFC (bottom)

development. The light blue shadows mark the position of regulatory elements. These two elements show species-specific sequences between humans and rhesus monkeys. The black arrow indicates the direction of *CBLN2* transcription.

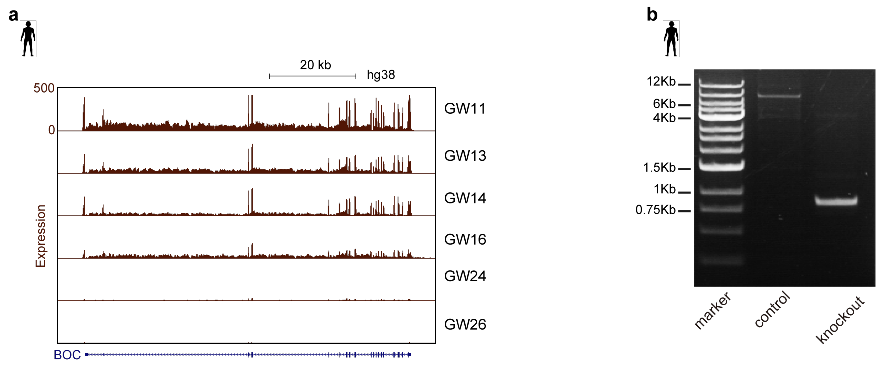

Supplementary Figure 11 | *BOC* enhancer knockout. **a**, Genome browser view of gene expression of *BOC* during human PFC development. **b**, The DNA electrophoretic gel image showing the sizes of PCR products across the *BOC* enhancer in *BOC* enhancer knockout and control human cortical organoids. Representative images are shown from n = 3 independent replicates.

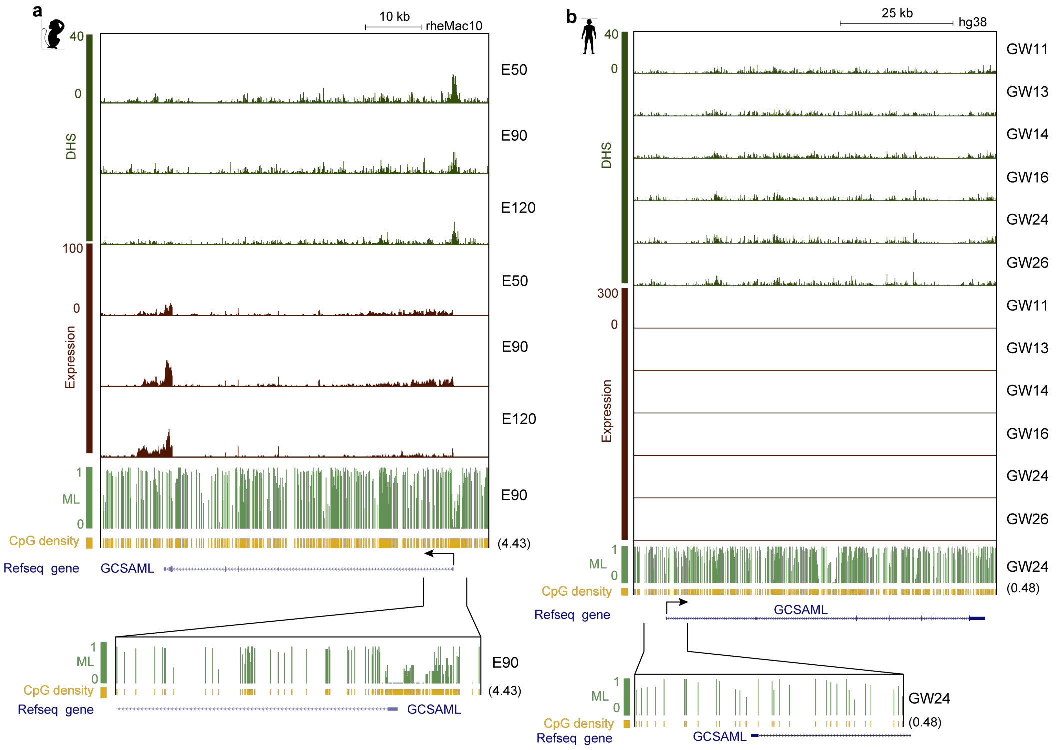

Supplementary Figure 12 | CpG densities and DNA methylation levels of *GCSAML*. **a**, Genome browser view of promoter chromatin accessibility, expression level, methylation levels and CpG densities of *GCSAML* during rhesus PFC development. The numbers in parentheses indicate the promoter CpG densities. **b**, Genome browser view of promoter chromatin accessibility, expression level, methylation levels and CpG densities of *GCSAML* during human PFC development. The numbers in parentheses indicate the promoter CpG densities.

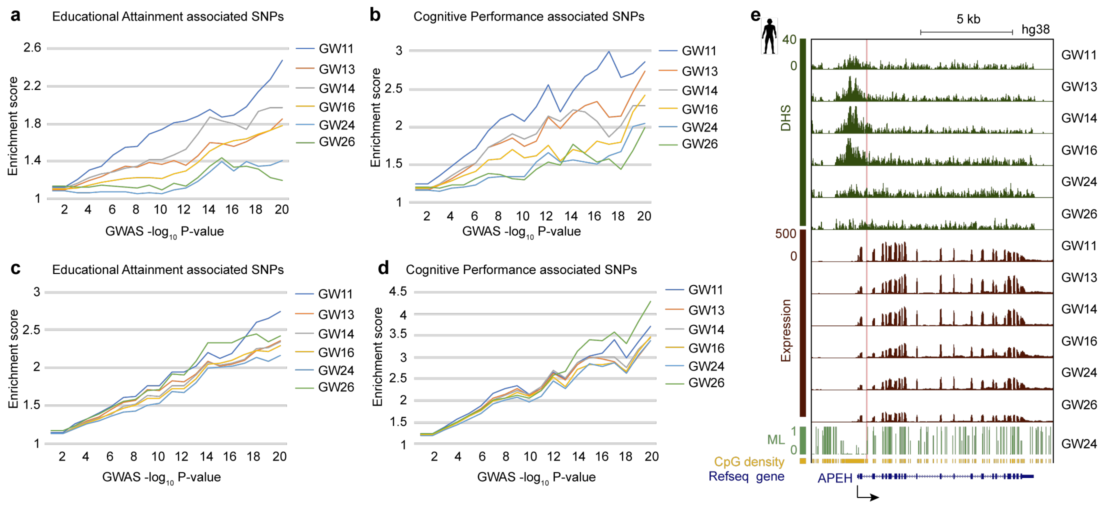

Supplementary Figure 13 | Enrichment of educational attainment or cognitive performance associated SNPs in the open chromatin regions in the human PFC. **a**, Plots showing enrichment scores of educational attainment associated SNPs in DHS regions at different PFC stages. The x axis represents the strength of the association between SNP and educational attainment. **b**, Plots showing enrichment scores of cognitive performance associated SNPs in DHS regions at different PFC stages. The x axis represents the strength of the association between SNP and cognitive performance. **c**, Plots show enrichment scores of educational attainment-associated SNPs in conserved DHSs in human PFCs at different developmental stages. **d**, Plots show enrichment scores of cognitive performance-associated SNPs in the conserved DHS regions at different PFC stages. **e**, Genome browser view of promoter chromatin accessibility and RNA expression levels of *APEH* in human PFCs at different stages. Pink lines represent the location of a SNP which is associated with both educational attainment and cognitive performance.

Supplementary Table 1 | The number of *cis*-elements and genes in each cluster of rhesus PFC.

	Elements number	Gene number
Cluster 1	14,715	1,293
Cluster 2	10,714	997
Cluster 3	3,075	283
Cluster 4	1,277	149
Cluster 5	6,773	506
Cluster 6	967	102

Supplementary Table 2 | The number of *cis*-elements and genes in each cluster of human PFC.

	Elements number	Gene number
Cluster 1	57,313	803
Cluster 2	38,186	916
Cluster 3	12,619	449
Cluster 4	6,814	336
Cluster 5	7,457	369
Cluster 6	6,016	350
