# Supplementary material for: Comparison of chromatin accessibility landscapes during early development of prefrontal cortex between rhesus macaque and human
Source: Nat Commun. 2022 Jul 6;13:3883. doi: 10.1038/s41467-022-31403-3 (PMC9259620; doi:10.1038/s41467-022-31403-3)
Supplement: Supplementary file 2 — Description of Additional Supplementary Files [file 41467_2022_31403_MOESM2_ESM.pdf]

Description of Additional Supplementary Files

File Name: Supplementary Data 1
Description: GO enrichment of genes in different clusters of Figure 1c. P-values are calculated based on the accumulative hypergeometric distribution.

File Name: Supplementary Data 2
Description: Species specific or conserved orthologous genes between rhesus cluster 1 and human cluster 1.

File Name: Supplementary Data 3
Description: Species specific or conserved orthologous genes between rhesus cluster 2 and human cluster 2.
