# Supplementary material for: Comparison of chromatin accessibility landscapes during early development of prefrontal cortex between rhesus macaque and human
Source: Nat Commun. 2022 Jul 6;13:3883. doi: 10.1038/s41467-022-31403-3 (PMC9259620; doi:10.1038/s41467-022-31403-3)
Supplement: Supplementary file 6 — Reporting Summary [file 41467_2022_31403_MOESM6_ESM.pdf]

nature research

Corresponding author(s): Xingxu Huang, Xiaoqun Wang and Jiang Liu

Last updated by author(s): Mar 26, 2022

Reporting Summary

Nature Research wishes to improve the reproducibility of the work that we publish. This form provides structure for consistency and transparency in reporting. For further information on Nature Research policies, see our Editorial Policies and the Editorial Policy Checklist.

Statistics

For all statistical analyses, confirm that the following items are present in the figure legend, table legend, main text, or Methods section.

n/a	Confirmed	
☐	☒	The exact sample size (*n*) for each experimental group/condition, given as a discrete number and unit of measurement
☐	☒	A statement on whether measurements were taken from distinct samples or whether the same sample was measured repeatedly
☐	☒	The statistical test(s) used AND whether they are one- or two-sided<br>*Only common tests should be described solely by name; describe more complex techniques in the Methods section.*
☐	☒	A description of all covariates tested
☐	☒	A description of any assumptions or corrections, such as tests of normality and adjustment for multiple comparisons
☐	☒	A full description of the statistical parameters including central tendency (e.g. means) or other basic estimates (e.g. regression coefficient) AND variation (e.g. standard deviation) or associated estimates of uncertainty (e.g. confidence intervals)
☐	☒	For null hypothesis testing, the test statistic (e.g. *F*, *t*, *r*) with confidence intervals, effect sizes, degrees of freedom and *P* value noted<br>*Give P values as exact values whenever suitable.*
☒	☐	For Bayesian analysis, information on the choice of priors and Markov chain Monte Carlo settings
☒	☐	For hierarchical and complex designs, identification of the appropriate level for tests and full reporting of outcomes
☐	☒	Estimates of effect sizes (e.g. Cohen's *d*, Pearson's *r*), indicating how they were calculated

Our web collection on statistics for biologists contains articles on many of the points above.

Software and code

Policy information about availability of computer code

Data collection	No computer code were used to collect the data.
Data analysis	The low quality bases in the reads of DNase-seq data were removed and the reads were cropped to 36 bp by Trimmomatic v0.33. Only read1 were used for mapping. Human libraries were aligned to hg38 and rhesus libraries were aligned to RheMac10 by Bowtie v1.2.0 with parameter " -m 1". Samtools v1.3.1 was used to remove low mapping quality reads (MAPQ < 10) and Picard v2.5.0 was used to remove PCR duplicated reads. To exclude the effect of sequencing depth, we sorted the alignment file by reads name for each replicate, then extracted the first 20 million reads for further analysis. DHSs were called by hotspot algorithm v4.1 with FDR < 0.01. Only DHSs with P values less than 1e-5 were retained. The UCSC "liftOver" tool was used to convert the coordinates of genomic regions between rhesus and human or between different genome versions in rhesus or human. Bedtools v2.26.0 was used for genomic annotation. For human RNA-seq data, we mapped the paired-end reads to hg38 using hisat v2.0.4 with parameter "-dta-cufflinks". For rhesus data, paired-end reads were aligned to rheMac10 by hisat v2.0.4 with parameter "-dta-cufflinks". Cufflinks v2.2.1 was used to calculate the FPKM of each gene. DNase-seq and RNA-seq tracks visualized in UCSC Genome Browser were generated by bamCoverage in Deeptools v2.5.7 suit with parameter "—normalizeUsing RPKM". For WGBS data, low quality reads were removed by Trimmomatic v0.33. For human WGBS data, paired-end reads were mapped to hg38 by Bismark v0.7 with default parameters. For rhesus WGBS data, paired-end reads were mapped to rheMac10 by Bismark v0.7 with default parameters. PCR duplicates were removed by Picard v2.5.0 and overlapped parts of pair-end reads were trimmed from one end by bamUtil v1.0.13. The methylation level of each CpG site was calculated by a custom script. Homer software v4.11 was used to perform motif analysis with default parameters. GO analysis was performed by Metascape web tool v3.5 with default parameters. The TissueEnrich package v1.14.0 was used to identify tissue-specifically expressed genes and broadly expressed genes. Statistical analysis and plotting were performed with R v3.6.1.<br>The codes used in this study were available if requested.

For manuscripts utilizing custom algorithms or software that are central to the research but not yet described in published literature, software must be made available to editors and reviewers. We strongly encourage code deposition in a community repository (e.g. GitHub). See the Nature Research guidelines for submitting code & software for further information.

Data

Policy information about availability of data

All manuscripts must include a data availability statement. This statement should provide the following information, where applicable:

- Accession codes, unique identifiers, or web links for publicly available datasets
- A list of figures that have associated raw data
- A description of any restrictions on data availability

The datasets generated and analysed during the current study are available in the Genome Sequence Archive under accession numbers CRA002225 [https://ngdc.cncb.ac.cn/gsa/browse/CRA002225] and HRA002180 [https://ngdc.cncb.ac.cn/gsa-human/browse/HRA002180]. The ChIP-seq data for human and rhesus forebrain analyzed in this study were downloaded from NCBI Gene Expression Omnibus (GEO) under accession GSE63649 [https://www.ncbi.nlm.nih.gov/geo/query/acc.cgi?acc=GSE63649].
The sequences and genomic annotations of reference genomes for human hg38/GRCh38 and rhesus rheMac10/Mmul_10 were downloaded from Ensembl (hg38 genome sequence [http://ftp.ensembl.org/pub/release-104/fasta/homo_sapiens/dna/Homo_sapiens.GRCh38.dna.primary_assembly.fa.gz], hg38 annotation [http://ftp.ensembl.org/pub/release-104/gtf/homo_sapiens/Homo_sapiens.GRCh38.104.chr.gtf.gz], rheMac10 genome sequence [http://ftp.ensembl.org/pub/release-104/fasta/macaca_mulatta/dna_index/Macaca_mulatta.Mmul_10.dna.toplevel.fa.gz], rheMac10 annotation [http://ftp.ensembl.org/pub/release-104/gtf/macaca_mulatta/Macaca_mulatta.Mmul_10.104.chr.gtf.gz]).
The SNPs associated with educational attainment or cognitive performance were downloaded from https://www.thessgac.org/data. The information of SNPs in human genome was downloaded from http://ftp.1000genomes.ebi.ac.uk/vol1/ftp/release/20130502/. The expression levels of genes in human various tissues were downloaded from GTEx Portal V8 [https://storage.googleapis.com/gtex_analysis_v8/rna_seq_data/GTEx_Analysis_2017-06-05_v8_RNASeQCv1.1.9_gene_tpm.gct.gz]. The interaction profile for enhancers in human cerebral cortex at the mid-gestation stages (GW17-GW18 stages) were obtained from the supplementary information files in a published article [https://static-content.springer.com/esm/art%3A10.1038%2Fnature19847/MediaObjects/41586_2016_BFnature19847_MOESM87_ESM.xlsx].

Field-specific reporting

Please select the one below that is the best fit for your research. If you are not sure, read the appropriate sections before making your selection.

☒ Life sciences ☐ Behavioural & social sciences ☐ Ecological, evolutionary & environmental sciences

For a reference copy of the document with all sections, see nature.com/documents/nr-reporting-summary-flat.pdf

Life sciences study design

All studies must disclose on these points even when the disclosure is negative.

Sample size	No sample size calculation was performed. In total, Six human embryos and six rhesus monkey embryos were used in this study. For rhesus PFC samples, two rhesus monkey embryos were used for each stage. Due to the scarcity of human embryo samples, only one human embryo was used for each stage, but two technical replicates were carried out for human samples at each stage. The data with high correlation coefficients (Pearson's correlation coefficients > 0.80) between two replicates were used for further analysis. For DNase-seq data, the two-replicate guideline was suitable for the identification of DHSs, which is a binary determination.
Data exclusions	For DNase-seq, in order to exclude the effect of sequencing depth, we sorted the alignment file by reads name for each replicate, then extracted the first 20 million reads for further analysis.
Replication	For rhesus, we performed 2 biological replicates for each stage of embryos. The two biological replicates show high level of Pearson's Correlation Coefficient (r > 0.80) for each stage of samples. For human, we construct two sequencing libraries for each stage of embryo. The two libraries show high level of Pearson's Correlation Coefficient (r > 0.80) for each stage.<br>All attempts at replication were successful for rhesus and human experiments.
Randomization	The allocation of samples is random.
Blinding	The authors were not blinded to group allocation during sample collection or analysis, as the information on materials was essential for the experiment design and analysis.

Reporting for specific materials, systems and methods

We require information from authors about some types of materials, experimental systems and methods used in many studies. Here, indicate whether each material, system or method listed is relevant to your study. If you are not sure if a list item applies to your research, read the appropriate section before selecting a response.

Materials & experimental systems

n/a	Involved in the study
☐	☒ Antibodies
☐	☒ Eukaryotic cell lines
☒	☐ Palaeontology and archaeology
☐	☒ Animals and other organisms
☐	☒ Human research participants
☒	☐ Clinical data
☒	☐ Dual use research of concern

Methods

n/a	Involved in the study
☒	☐ ChIP-seq
☒	☐ Flow cytometry
☒	☐ MRI-based neuroimaging

Antibodies

Antibodies used

rabbit anti-PAX6 (901301, BioLegend)
mouse anti-TUBB3 (801201, BioLegend)
Alexa Fluor 488 conjugated donkey anti-rabbit (ab150073, abcam)
Alexa Fluor 594 conjugated donkey anti-mouse secondary antibodies (ab150108, abcam)

Validation

The application of these primary antibodies for immunostaining was validated by the manufacturers or published works. This information was available on the websites for these antibody reagents. For example, rabbit anti-PAX6 antibody can be used for immunostaining in human cells, which is validated by Quadrato G, et al. 2017. Nature (PMID:28445462). Mouse anti-TUBB3 antibody is applicable for immunotaining in human cells, which is validated by the manufacturers and published works, such as Rajesh Ambasudhan et al. 2011. Cell Stem Cell (PMID: 21802386).

Eukaryotic cell lines

Policy information about cell lines

Cell line source(s)

human H9 embryonic stem cells are from ATCC

Authentication

The cell lines used in the study were not authenticated by ourselves. Human H9 cell lines were validated by their morphology, gene expression patterns and organoid formation.

Mycoplasma contamination

All cell lines used in this study have tested negative for mycoplasma contamination

Commonly misidentified lines
(See ICLAC register)

No commonly misidentified cell lines were used in the study

Animals and other organisms

Policy information about studies involving animals; ARRIVE guidelines recommended for reporting animal research

Laboratory animals

The rhesus macaque (Macaca mulatta) at embryo day 50, embryo day 90 and embryo day 120 in healthy status were used in this study. The sex of macaque embryos was not determined.

Wild animals

No wild animals were used.

Field-collected samples

No field collected samples

Ethics oversight

All monkey experiment procedures were approved by the Ethics Committee of the Institute of Zoology and Kunming Institute of Zoology, Chinese Academy of Sciences and the Ethics Committee of the Beijing Institute of Genomics. All the experiments in this study are in compliance with these relevant ethical regulations.

Note that full information on the approval of the study protocol must also be provided in the manuscript.

Human research participants

Policy information about studies involving human research participants

Population characteristics

The donors of human samples are pregnant women who could not continue pregnancy because of their own disease (such as cervical insufficiency, heart disease, inevitable abortion, eclampsia, etc. in China. The information of donors' ages was not obtained due to the privacy protection. All embryo and fetal tissues were between 11-26 gestational weeks. Gestational age was measured in weeks from the first day of the woman's last menstrual cycle to the sample collecting date. The gender of human embryos was not determined.

Recruitment

Beijing Anzhen Hospital was in charge of recruiting donors for this research. The patients decided to have an abortion first, and then they were asked whether they would agree to donate the fetal tissues to this study. The de-identified human fetal tissue samples were collected after the donor patients signing informed consent document. No compensation was offered for the donors.

Ethics oversight	The de-identified human tissue collection and research protocols were approved by the Reproductive Study Ethics Committee of Beijing Anzhen Hospital and the institutional review board (ethics committee) of the Institute of Biophysics and the Ethics Committee of the Beijing Institute of Genomics,Chinese Academy of Sciences.

Note that full information on the approval of the study protocol must also be provided in the manuscript.
